# ZmSKIP enhances drought tolerance by reducing stomatal aperture in maize

**DOI:** 10.1371/journal.pgen.1012077

**Published:** 2026-04-13

**Authors:** Yao Wang, Yujiao Zhou, Qimeng Li, Yanyan Zhang, Li Yang, Qiao Zhou, Wen Xu, Tianhong Liu, Yaxi Liu, Fengkai Wu, Guangchao Sun, Wei Guo, Yanli Lu, Jie Xu

**Affiliations:** 1 State Key Laboratory of Crop Gene Exploration and Utilization in Southwest China, Wenjiang, Sichuan, China; 2 Maize Research Institute of Sichuan Agricultural University, Wenjiang, Sichuan, China; 3 Sichuan Tianfu New Area Rural Revitalization Research Institute, Tianfu New Area, China; 4 Triticeae Research Institute, Sichuan Agricultural University, Wenjiang, Sichuan, China; Max Planck Institute of Molecular Plant Physiology: Max-Planck-Institut fur molekulare Pflanzenphysiologie, GERMANY

## Abstract

SKI-INTERACTING PROTEINS (SKIPs), primarily known as splicing factors, control gene expression at the post-transcriptional level in stress responses in plants. However, little is known about SKIPs in regulating plant drought stress at the transcriptional level, particularly in maize (*Zea mays L.*). Here, we discover that ZmSKIP enhances drought tolerance in maize. *ZmSKIP* transgenic plants were generated to study how ZmSKIP positively regulates drought tolerance. Overexpression of *ZmSKIP* promoted stomatal closure and reduced water loss, whereas the opposite effect was observed in *skip-aa* mutants. ZmSKIP directly binds to the “TAATA” motif in the promoter of B-cell lymphoma 2-associated athanogene 8 (*ZmBAG8*). *bag8* mutants exhibit the decreased water loss and reduced stomatal aperture phenotype under drought stress. Additionally, ZmSKIP can be recruited by ZmBAG8 in stress granules (SGs) to decrease its protein abundance in the nucleus. Increased *ZmBAG8* expression leads to larger stomatal aperture and normal plant growth. Under drought stress, the interaction between ZmSKIP and ZmBAG8 was abolished, while ZmSnRK2.3 phosphorylates ZmSKIP at Ser^236^ and Ser^244^ to enhances drought tolerance by strengthening the ability of ZmSKIP to suppress *ZmBAG8* expression. Thus, our findings demonstrate that ZmSnRK2.3-mediated phosphorylation of ZmSKIP reduces *ZmBAG8* expression and stomatal aperture, thereby enhancing drought tolerance in maize.

## Author summary

As dual-function factors, SKIPs mainly regulate gene expression at the transcriptional and post-transcriptional levels in stress responses in plants. However, little is known about SKIPs in regulating plant drought stress at the transcriptional level in maize (*Zea mays L.*). Here, we discover that ZmSKIP enhances drought tolerance by promoting stomatal closure and reducing water loss in maize. ZmSKIP directly binds to the “TAATA” motif in the promoter of *ZmBAG8*. *bag8* mutants exhibit the decreased water loss and reduced stomatal aperture phenotype under drought stress. Additionally, ZmSKIP can be recruited by ZmBAG8 in stress granules (SGs) to decrease its protein abundance in the nucleus. Increased *ZmBAG8* expression leads to larger stomatal aperture and normal plant growth. Under drought stress, the interaction between ZmSKIP and ZmBAG8 was abolished, while ZmSnRK2.3 phosphorylates ZmSKIP at Ser^236^ and Ser^244^ to enhances drought tolerance by strengthening the ability of ZmSKIP to suppress *ZmBAG8* expression. Thus, our findings demonstrate that ZmSnRK2.3-mediated phosphorylation of ZmSKIP reduces *ZmBAG8* expression and stomatal aperture, thereby enhancing drought tolerance in maize.

## Introduction

Maize (*Zea mays L*.) is one of the most economically significant crop for food, feed, and fuel production and exhibits strong adaptability and wide cultivation areas worldwide [1]. However, abiotic stresses, particularly drought, are major limiting factors of maize yield. To cope with adverse conditions, plants have evolved various physiological and biochemical mechanisms to enhance their resistance to abiotic stresses, thereby improving yield stability [2]. One of the most critical strategies used by plants is stomatal regulation, such as stomatal aperture and stomatal patterning, which helps optimize water consumption and improve survival under drought stress [3–5]. Stomatal aperture is controlled by the turgor pressure of guard cells, which is regulated by transmembrane transporters and channels, including K^＋^ and anion channels [6–9] When plants suffer from drought stress, the reduced stomatal aperture helps maintain the dynamic balance between leaf transpiration and photosynthesis, which in turn improves plant survival. Therefore, understanding the molecular mechanisms governing stomatal aperture is crucial for the development of drought-resistant and high-yield crop varieties [8,10–13]. In this study, we demonstrated that a Ski-interacting protein (SKIP) positively regulates drought tolerance by controlling stomatal aperture to improve maize yield.

SKIP is a class of conserved proteins characterized by an SNW/SKIP domain that contains an S-N-W-K-N peptide signature. These proteins play fundamental roles as splicing and transcription factors (TFs) that regulate gene expression at both the transcriptional and post-transcriptional levels [14–18]. In plants, SKIP primarily functions as a splicing factor by integrating into the spliceosome and interacting with spliceosome components, such as MOS4-Associated Complex 3 (MAC3), Spliceosomal Timekeeper Locus 1 (STIPL1), and Pleiotropic Regulatory Locus 1 (PRL1) [19,20,21]. Beyond its role in splicing, SKIP regulates the circadian clock and salt stress response in *Arabidopsis* by modulating alternative pre-mRNA splicing [16,22]. Additionally, SKIP interacts with the polymerase-associated factor 1 complex (Paf1c), a transcriptional activation complex that mediates flowering by influencing the splicing and histone modification of Flowering Locus C (FLC) [23–25]. Notably, other spliceosome components, such as MAC3, STIPL1, and PRL1, were dispensable for *FLC-*mediated flowering regulation. In *Arabidopsis*, SKIP integrates into two distinct complexes to regulate the floral transition and stress responses at the transcriptional and post-transcriptional levels, respectively [20]. The interaction between SKIP and Shoot Meristemless (STM) is crucial for controlling the expression of a similar set of genes that ultimately mediate the development of the Shoot Apical Meristem (SAM) [26]. Under cold stress, SKIP directly interacts with C-REPEAT BINDING FACTORs (CBFs) proteins to facilitate its nuclear condensates formation, which positively regulates acquired freezing tolerance [27]. Furthermore, SKIP functions as a TF in the abscisic acid signaling pathway, enhancing salt and osmotic tolerance through alternative splicing [28,29]. In Arabidopsis, the expression of *SKIP* is induced by ABA, NaCl and mannitol. Overexpression of *SKIP* enhances the tolerance to salt stress [28]. In rice, SKIP promotes cell viability and drought tolerance by interacting with Cyclophilin 18–2 (CYP18–2) [30,31]. Collectively, SKIP is involved in all aspects of plant growth and development, spanning both transcriptional and post-transcriptional regulation. However, the detailed regulatory mechanisms of SKIP as a TF in maize, particularly under drought stress, remain unclear.

B-cell lymphoma 2 (Bcl-2)-associated athanogen (BAG) proteins belong to a protein family associated with drought stress. BAG is evolutionarily conserved and functions as a multifunctional co-chaperone that can flexibly interact with a variety of proteins and regulate various processes, from growth and development to stress responses [32,33,34]. BAG proteins were first identified in animals [32]. Studies have demonstrated that BAGs act as molecular switches for multiple targets to maintain metabolic homeostasis and are involved in several biological processes, including apoptosis, tumor formation, stress response, and the cell cycle [35]. However, studies investigating the BAG family in plants are relatively scarce and have primarily focused on the model plant Arabidopsis. In plants, the BAG protein family can be classified into two groups based on their structural characteristics [36]. The first group contains a ubiquitin-like (UBL) domain at the N-terminus, similar to the structural composition of animal BAG1. The second group includes plant-specific CaM-binding IQ motifs located adjacent to the BAG domain [37]. Recent studies have demonstrated that BAGs play key roles in plant growth, autophagy, and stress responses (e.g., heat, cold, drought, and biotic stress) [33,38–40]. In Arabidopsis, the seven *BAG* homologs can be classified into two groups based on their structural characteristics [36,41]. AtBAG7 interacts with basic leucine zipper 28 (AtbZIP28) under normal conditions. However, endoplasmic reticulum (ER) stress triggers the proteolytic release of AtBAG7, which is then SUMOylated and translocated to the nucleus, where it interacts with AtWRKY29 to regulate stress-responsive genes [24]. Similar to heat stress, ER stress is triggered when Phytophthora PAMPs are infected [42]. BAG7 binds to WRKY29 to regulate ER stress-mediated plant immunity, and is directly activated by bZIP28. AtBAG2 and AtBAG6 may negatively regulate drought tolerance by influencing the expression of stress-related genes such as RD29A/B, NCED3, and ABI in Arabidopsis [43]. Recently, *SlBAG9* overexpression in Arabidopsis was shown to increase sensitivity to drought stress. This reduced tolerance may be due to SlBAG9-mediated downregulation of stress-related gene expression and severe oxidative damage [44]. However, the detailed mechanisms of action of BAG proteins in maize remain unclear.

In this study, we demonstrate that ZmSKIP positively regulates drought tolerance in maize by modulating stomatal aperture. As a TF, ZmSKIP suppresses the expression of *ZmBAG8,* which negatively affects drought tolerance by influencing the stomatal aperture in maize. Additionally, ZmBAG8 interacts with ZmSKIP within stress granules (SGs), reducing nuclear ZmSKIP protein levels, thereby ensuring proper stomatal aperture and normal maize growth. Notably, ZmSKIP is phosphorylated by ZmSnRK2.3, which enhances the inhibition of *ZmBAG8* expression under drought stress conditions. Collectively, ZmSKIP plays a crucial role in the drought response, and these findings offer a promising strategy for enhancing drought resistance in maize.

## Results

### ZmSKIP positively regulates drought tolerance in maize

In maize, the rate of water loss (RWL) from detached leaves is an important phenotype associated with drought tolerance [45–47]. We analyzed this phenotype (10 h *in vitro*) in 98 inbred maize lines at the three-leaf stage. Based on the survival rates of drought-stressed plants and the classification of drought tolerance in inbred maize [45,46], we observed that inbred lines with better drought tolerance (indicated by higher survival rates under drought conditions) exhibited lower RWL in the detached leaves (S1a Fig and S1 Data). A significant negative correlation was observed between the drought survival rate and RWL of detached leaves (S1b Fig, Pearson correlation, *p* = 0.036). To identify candidate genes regulating the RWL, we performed a generalized linear model (GLM) regression analysis to associate gene expression levels in seedling leaves under drought stress with the RWL in detached leaves. Out of 44,117 expressed genes, 1,451 genes were significantly associated with RWL after multiple testing correction (Benjamini-Hochberg adjusted FDR < 0.01). To further prioritize genes with strong phenotypic contributions and robust expression patterns, we applied a stringent multi-step filtering strategy with: (1) explained a substantial proportion of phenotypic variance (*R*^2^ > 0.15); (2) exhibited highly significant differential expression between well-watered (WW) and water-stressed (WS) conditions among different maize inbred lines (Student’s *t*-tes*t* with Benjamini-Hochberg adjusted FDR < E^-10^); and (3) showed stable expression changes across the population (standard deviation (SD) of the expression difference WS-WW for each gene, SD < 10 across all inbred lines). This screening resulted in a final set of 99 high-confidence candidate genes.

Gene Ontology (GO) enrichment analysis of these 99 candidates revealed significant functional clustering in pathways related to transcriptional, mRNA splicing, chromatin DNA binding, and post-transcriptional regulation etc (S1c Fig). To further explore the transcriptional regulation of the identified candidate genes under drought, we performed a hierarchical clustering analysis of their expression levels (FPKM) in the association panel seedling leaves under both WW and WS conditions. The heatmap reveals distinct expression patterns across the population, with genes grouping into clusters based on their differential responses to drought stress (S1d Fig). Notably, Zm00001d052186 (*ZmSKIP*) exhibited a unique and striking expression profile, clustering into a separate branch distinct from the majority of other candidates. While most genes showed moderate to low expression variability, *ZmSKIP* maintained a distinct high-expression pattern that set it apart in the clustering tree. Functional annotation indicates that SKIP is both a key component of the spliceosome and transcription factor [27]*,* which is consistent with the results of the GO enrichment analysis. Correlation analysis between the measured RWL and *ZmSKIP* gene expression levels in leaves, using gene expression data from Sun et al. [47], indicated a significant negative correlation under drought stress (S1e Fig; Pearson’s correlation, *p* = 6.1e^-5^). The subcellular localization results showed that ZmSKIP was primarily expressed in the nucleus (S1f Fig). Taken together, given its distinctive expression pattern and its potential pivotal role in splicing and transcription, we focused on this gene for further functional validation and mechanistic dissection.

**Fig 1:**
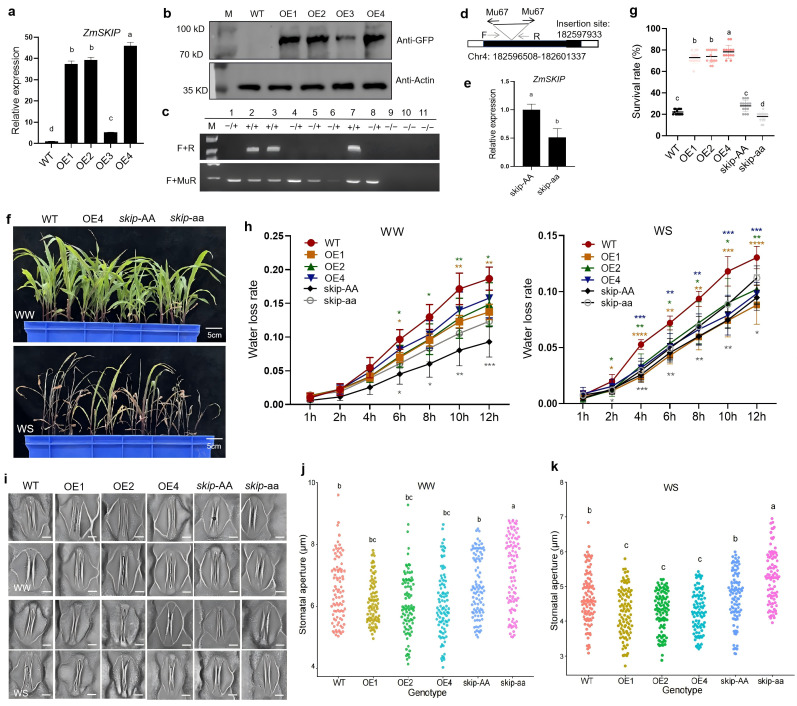
Phenotypic analysis of *ZmSKIP*-overexpressing (OE) transgenic maize and *skip* mutant plants at the seedling stage. a. Real-time qPCR analysis of the expression of *ZmSKIP* in *ZmSKIP*-OE plants. b. Western blot analysis of the protein level of ZmSKIP in *ZmSKIP*-OE plants by Anti-GFP. Actin was used as control. c. PCR analysis for *skip Mu* insertion inheritability. “**+/+**”, “**−/+**”, and “**−/−**” represent no insertion, heterozygous insertion, and homozygous insertion, respectively. d. A schematic indicates the transposon insertion site in *skip-aa* mutants. e. Real-time qPCR analysis of the expression of *ZmSKIP* in *skip Mu* mutants. f. Phenotypic analysis of *ZmSKIP* transgenic maize at the seedling stage under drought stress. g. Measurement and statistical analysis of the survival rate of *ZmSKIP* transgenic maize at the seedling stage under drought stress. h. Measurement and statistical analysis of the leaf water loss rate of *ZmSKIP* transgenic maize at the seedling stage under drought stress. i. Phenotypic analysis of the stomatal aperture of *ZmSKIP* transgenic maize at the seedling stage under drought stress. Bar = 10 μm. j. Measurement and statistical analysis of the stomatal aperture of *ZmSKIP* transgenic maize at the seedling stage under well-watered condition (n ≥ 50). k. Measurement and statistical analysis of the stomatal aperture of *ZmSKIP* transgenic maize at the seedling stage under drought stress (n ≥ 50). WT, wild type, KN5585; OE, overexpressing lines; *skip-aa*, *skip* homozygous *Mu* mutants; *skip-AA*, *skip-aa* controls (B73); WW, well-watered; WS, water-stressed (the soil water content <8%) at the seedling stage. *t-*t*est, with P < 0.05, **t-tes*t*, with P < 0.01, ***t-test, wi*t*h P < 0.001, ****t-test, with P < 0.0001. Differen*t* letters show significant differences (P < 0.05) between each other.

To further clarify the significance of ZmSKIP in maize drought tolerance, we generated *ZmSKIP*-overexpressing (OE) transgenic plants, in which the full-length open reading frame (ORF) of *ZmSKIP* fused with GFP and FLAG tags was constitutively expressed in the pCAMBIA3301 vector which under the control of the maize ubiquitin promoter. *skip Mutator* (*Mu*) insertion mutant lines were employed at http://chinamu.jaas.ac.cn/content/about.html. We obtained four independent transgenic lines, among which three T2 OE lines (OE1, OE2, and OE4) with higher *ZmSKIP* expression levels (Fig 1a-1b) and *skip Mu* homozygous insertion mutants (*skip*-aa) with lower transcript levels were selected for phenotypic analyses (Fig 1c-1e). Notably, no significant growth differences were observed under normal (well-watered, WW) conditions; however, drought tolerance was significantly improved in *ZmSKIP*-OE transgenic plants under water-stressed (WS, soil water content < 8%), with a higher plant survival rate (Fig 1f-1g). We found that the *ZmSKIP*-OE transgenic plants exhibited lower water loss rate at the seedling stage, which was opposite to *skip-aa* (Fig 1h). Stomata are the main pores that allow the exchange of water and air in plant leaves, and stomatal aperture is essential for water retention and efficient photosynthesis [13]. Although the stomatal densities of the different lines were similar (S2a-S2b Fig), the stomatal aperture of *ZmSKIP*-OE was significantly narrower, whereas that of *skip-aa* was broader than that of the controls under drought stress (Fig 1i-1k and S2 Data). However, no variation was observed in the root-shoot ratio of *ZmSKIP*-OE and mutant plants relative to that of the controls (WT and *skip*-AA) (S2c-S2d Fig). *ZmSKIP*-OE plants exhibited a remarkable decrease in leaf ion permeability, in contrast to the mutant plants, which exhibited an increase in ion permeability compared to the controls (S2e-S2f Fig).

**Fig 2:**
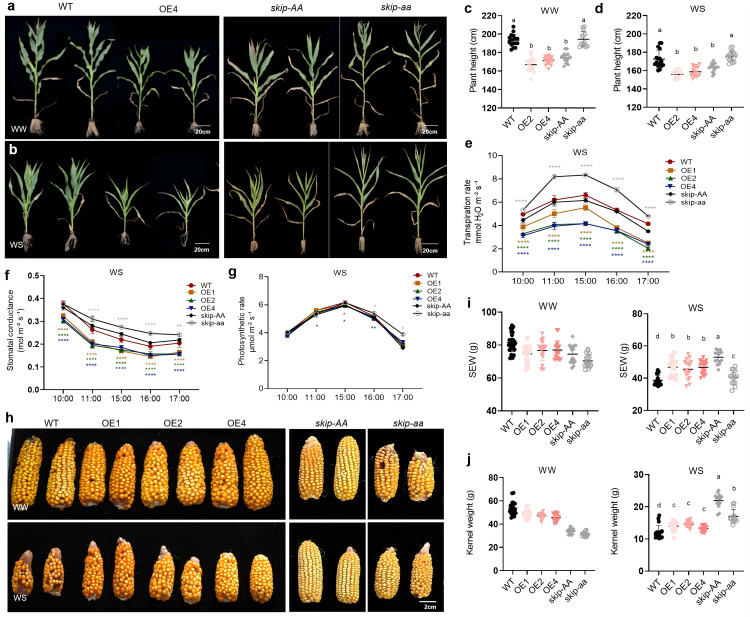
Phenotypic analysis of *ZmSKIP*-overexpressing (OE) transgenic maize and *skip* mutant plants at the adult stage in the field. a. Plant height analysis of *ZmSKIP* transgenic maize under well-watered (WW) conditions. b. Plant height analysis of *ZmSKIP* transgenic maize under water-stressed (WS) conditions. c. Measurement and statistical analysis of the plant height of *ZmSKIP* transgenic maize under WW conditions. d. Measurement and statistical analysis of the plant height of *ZmSKIP* transgenic maize under WS conditions. e. Measurement and statistical analysis of the transpiration rate of *ZmSKIP* transgenic maize at different times (10:00 am-17:00 pm) under WS conditions. f. Measurement and statistical analysis of the stomatal conductance of *ZmSKIP* transgenic maize at different times (10:00 am-17:00 pm) under WS conditions. g. Measurement and statistical analysis of the photosynthetic rate of *ZmSKIP* transgenic maize at different times (10:00 am-17:00 pm) under WS conditions. h.Ear phenotypic analysis of *ZmSKIP* transgenic maize. i. Measurement and statistical analysis of the single Ear Weight (SEW) of *ZmSKIP* transgenic maize (n ≥ 15). j. Measurement and statistical analysis of the kernel weight of *ZmSKIP* transgenic maize (n ≥ 15). WT, wild type, KN5585; OE, overexpressing; *skip-aa*, *skip* homozygous *Mu* mutants; *skip-AA*, *skip-aa* controls, B73; WW, well-watered; WS, water-stressed. *t-test, with P < 0.05, **t-*t*est, with P < 0.01, ****t-tes*t*, with P < 0.0001. Different letters show significant differences (P < 0.05) between each other.

Plant height, transpiration rate, stomatal conductance, photosynthetic rate and yield parameters, including Single Ear Weight (SEW) and kernel weight, were measured during the adult plant stage. The results showed that *ZmSKIP*-OE plants were shorter than WT plants, and *Mu* mutants were taller than controls under both WW and WS conditions (Fig 2a-2d). Under drought conditions, the transpiration rate, and stomatal conductance of *ZmSKIP*-OE plants were significantly lower than those of the WT, whereas the phenotype of *skip-aa* mutants was the opposite (Fig 2e-2f). Meanwhile, the photosynthetic rate did not change significantly, except for the OE4 at 11:00 am, 15:00 pm, 16:00 pm, and the *skip-aa* at 16:00 pm (Fig 2g). The yield of SEW and kernel weight of *ZmSKIP* OE plants were significantly increased (Fig 2h-2j and S3 Data). Plants under drought stress respond by promoting the biosynthesis of plant hormones, such as abscisic acid (ABA), and ethylene, which further induce stomatal closure through the activation of reactive oxygen species (ROS) and H_2_O_2_ [48]. As shown in S3a-S3b Fig, *ZmSKIP* expression increased in response to ABA and ethylene. Furthermore, NBT and DAB staining results indicated that the concentrations of ROS and O_2_^2-^ in *ZmSKIP* OE plants were significantly decreased, whereas the opposite was observed in *skip-aa Mu* plants compared with the controls (S3c-S3d Fig). Based on these results, we concluded that ZmSKIP may respond to drought stress via ABA-induced stomatal closure to reduce water loss and positively regulate drought tolerance in maize.

### ZmSKIP suppresses the expression of Zm*BAG8* by directly binding to its promoter via the “TAATA” motif

**Fig 3:**
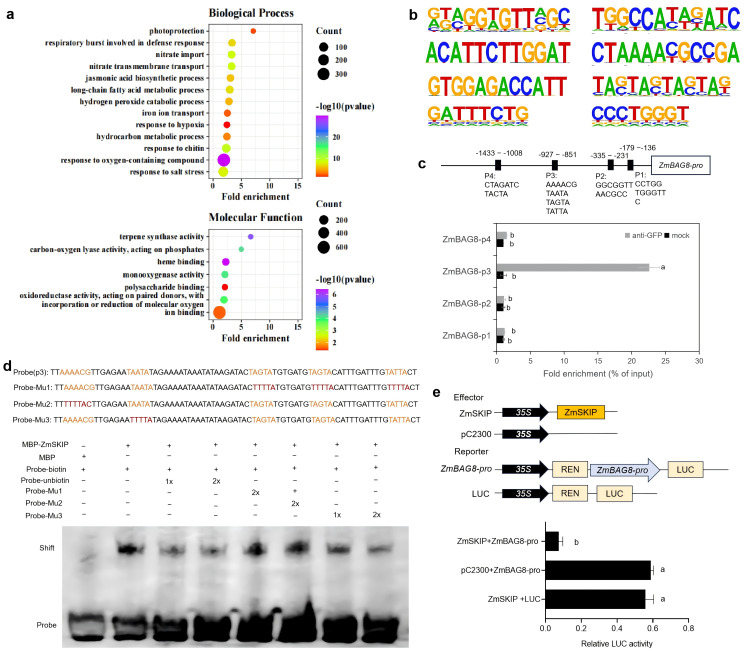
ZmSKIP negatively regulates *ZmBAG8* expression. a. *ZmSKIP*-OE (OE2) vs. WT plant DEGs enriched in Gene ontology (GO) scatterplot of biological processes by RNA-seq. b. Diagram of eight conserved motifs of ZmSKIP identified by CUT&Tag assays. The results were calculated using two biological replicates. c. ChIP-qPCR analysis of ZmSKIP binding to the *ZmBAG8* promoter. (upper) The horizontal line represents the *ZmBAG8* promoter; the black rectangles, which contain the conservative motifs below, represent conserved motifs in the promoter; p1 to p4 indicate the fragments detected by ChIP-qPCR. The numbers along the gene model are relative to the ATG. The ChIP signal is expressed as the percentage of immunoprecipitated DNA in the total input DNA. mock, ChIP without anti-GFP but with added lgG antibody as a negative control. d. EMSA of ZmSKIP binding to the conserved motifs in p3 of the *ZmBAG8* promoter. Biotin-labeled probes were incubated with MBP-ZmSKIP *in vitro*. Unlabeled probes were used for competition, and mutated biotin-labeled conserved motifs were used as negative controls. Normal (up, the orange letters indicate normal motifs in p3 of the *ZmBAG8* promoter) or mutated probes (down, the red letters indicate mutated motifs in p3 of the *ZmBAG8* promoter) are shown in d. e. Dual-LUC assay of ZmSKIP on *ZmBAG8* promoter activity in maize protoplasts. The relative LUC activities were normalized to the REN LUC reference. The mean value and SD are calculated from three biological replicates. Different letters show significant differences (P < 0.05) between each other.

To understand the molecular mechanism of ZmSKIP‐mediated stomatal closure in maize, we employed RNA‐seq assays to analyze the genes regulated by ZmSKIP from *ZmSKIP*-OE (OE2) and WT coleoptile libraries, respectively. RNA-seq analysis showed 2,081 differentially expressed genes (DEGs) (adjusted P-value < 0.05), including 1,171 upregulated and 910 downregulated DEGs, in the *ZmSKIP*-OE transgenic line compared to those in the WT (S4a Fig and S4 Data). Gene Ontology (GO) analysis indicated that these DEGs were primarily enriched for responses to oxygen-containing compounds, responses to hypoxia and hydrogen peroxide catabolic processes etc. Molecular function analysis showed that the DEGs mainly exhibited ion-binding activity (Fig 3a). *BAG* genes are associated with stomatal movement by interacting with KAT proteins to affect potassium ion fluidity [24,39,40]. Based on the main phenotype of stomatal aperture observed in the *ZmSKIP* transgenic plants under drought stress. One *BAG* gene (*ZmBAG8*, Zm00001d010727) was chosed among the identified DEGs for further study (S4 Data). Expression analysis indicated that *ZmBAG8* was expressed at higher levels in *skip-aa* mutants but at lower levels in *ZmSKIP*-OE transgenic plants than in the controls (WT and *skip*-AA) (S4b Fig). We therefore speculate that ZmSKIP may directly regulate *ZmBAG8* expression to influence the stomatal aperture in maize.

To investigate whether ZmSKIP directly regulates the expression of conserved motifs in *ZmBAG8*, we performed a CUT&Tag assay. ZmSKIP contains eight conserved motifs in its target genes (Fig 3b). Several possible ZmSKIP-binding elements (p1-p4) in the promoter were identified by analyzing the promoter sequence of *ZmBAG8*. ZmSKIP bound directly to the p3 segment of the *ZmBAG8* promoter, as revealed by a chromatin immunoprecipitation-quantitative polymerase chain reaction (ChIP-qPCR) assay based on the specificity of the GFP antibody (Fig 3c). Additionally, electrophoretic mobility shift assays (EMSAs) were performed to verify the interaction between ZmSKIP and the p3 segment of the *ZmBAG8* promoter *in vitro*. The results showed that strong signals were observed in the lanes with ZmSKIP protein and biotin‐labeled *cis*‐elements (Fig 3d). However, the intensity of shift bands was gradually reduced with increasing concentrations of unlabeled *cis*‐element competitors. When the conserved “AAAACG,” “TAGTA,” and “TATTA” *cis*-elements were mutated, the intensity of shift bands did not change. However, when the conserved “TAATA” *cis*-elements were mutated, the intensity of shift bands gradually decreased with increasing concentrations of mutant *cis*‐element competitors, indicating that ZmSKIP binds to the *ZmBAG8* promoter through the typical “TAATA” motif. Dual-luciferase (LUC) reporter assays in maize protoplasts showed that ZmSKIP suppressed the transcriptional activity of *ZmBAG8* (Fig 3e). Taken together, the above results demonstrated that ZmSKIP is capable of binding to the “TAATA” element of the promoter of *ZmBAG8* to suppress its expression in maize.

### ZmBAG8 negatively regulates drought tolerance in maize

To further explore the role of ZmBAG8 in drought stress in maize, the expression pattern of *ZmBAG8* and the correlation between drought traits in 368 drought breeds were identified. *ZmBAG8* mRNA accumulated at relatively high levels in the V1 leaf and R1 spike stalk, and different expression levels of this gene were detected in other tissues (S5a Fig). Correlation analysis indicated that *ZmBAG8* expression was negatively correlated with that in plants living under drought conditions. Under drought conditions, the higher the expression of *ZmBAG8*, the lower the survival rate of maize (S5b Fig). Additionally, under drought stress, *ZmBAG8* expressed at higher levels in *skip-aa* mutants but at lower levels in *ZmSKIP*-OE transgenic plants than in the controls (WT and *skip*-AA), which the overall expression level of *ZmBAG8* was lower than that under normal conditions (S4b Fig). These results suggested that ZmBAG8 negatively regulates drought tolerance in maize.

**Fig 4:**
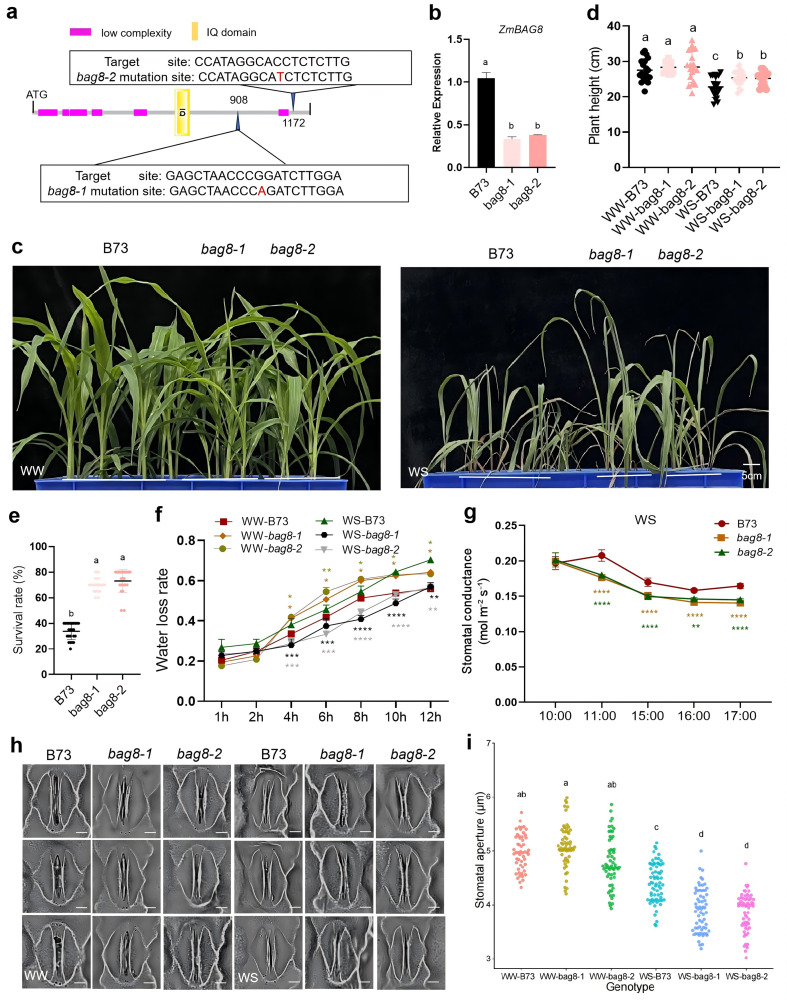
Phenotypic analysis of *bag8* EMS mutant maize. a. Diagram of conserved motifs of ZmBAG8 protein and EMS mutant sites identified in two *bag8* mutant plants. EMS4-3e5026 (*bag8-1*) and EMS4-3dee35 (*bag8-2*). **b.** qPCR analysis of the expression of *ZmBAG8* in two *bag8* homozygous mutants. c. Phenotypic analysis of *bag8* mutant maize at the seedling stage under well-watered (WW) and water-stressed (WS) conditions. Bar = 5 cm. d. Measurement and statistical analysis of plant height of *bag8* mutant maize at the seedling stage. e. Measurement and statistical analysis of survival rate of *bag8* mutant maize under drought stress condition at the seedling stage. f. Measurement and statistical analysis of the leaf water loss rate of *bag8* mutant maize at the seedling stage. g. Measurement and statistical analysis of the stomatal conductance of *bag8* mutant maize at different times (10:00 am-17:00 pm) under WS conditions. h. Phenotypic analysis of the stomata of *bag8* mutant maize at the seedling stage. Bar = 10 μm. i. Measurement and statistical analysis of the stomatal aperture of *bag8* mutant maize at the seedling stage (n ≥ 50). Bar = 10 μm. B73, control plants; WW, well-watered; WS, water-stressed (soil water content <8%) or 20% PEG6000 treatment at the seedling stage. *t-*t*est, with P < 0.05, **t-tes*t*, with P < 0.01, ***t-test, wi*t*h P < 0.001, ****t-test, with P < 0.0001. Differen*t* letters show significant differences (P < 0.05) between each other.

Two EMS mutants of *bag8* (http://elabcaas.cn/memd/public/indices/, No: EMS4-3e5026 (*bag8–1*) and EMS4–3dee35 (*bag8–2*)) were used to determine whether ZmBAG8 functions in drought tolerance in maize. We extracted DNA from *bag8* mutant plants, single-sequenced the EMS mutation sites, and analyzed *ZmBAG8* transcript levels in the leaves of *bag8* plants. The results indicated that base G was mutated to A (G-A) at position 908 of the CDS region of *ZmBAG8* in *bag8–1* mutants*.* Base C was mutated to T (C-T) at position 1172 of the CDS region of *ZmBAG8* in *bag8–2* mutants (Fig 4a), and *ZmBAG8* expression was significantly reduced in both *bag8* maize mutants compared to the controls (B73) (Fig 4b). We analyzed the drought tolerance of *bag8* homozygous mutant plants. Notably, significantly improved drought tolerance was observed in *bag8* mutants under WS (Fig 4c). Further studies showed that the plant height and seedling survival rate of *bag8* mutants were also significantly higher than that of B73 plants under WS conditions (Fig 4d-4e). To analyze the reasons for the higher drought tolerance of *bag8* mutants, we measured the water loss rate of the isolated leaves, and stomatal conductance at different times (10:00 am-17:00 pm) of *bag8* mutants. The results showed that *bag8* mutants exhibited lower rates of water loss than B73 plants under WS conditions (Fig 4f). The change in stomatal conductance of *bag8* mutants was similar to that observed in the water loss rate results (Fig 4g). Further analysis revealed that stomatal aperture slight increased in *bag8* plants under WW conditions. However, the stomatal aperture was significant lower in *bag8* mutant plants than in B73 plants under WS conditions (Fig 4h-4i and S5 Data). The drought tolerance phenotype of *bag8* mutants is consistent with that of the ZmSKIP-OE transgenic plants. Based on these above results, we conclude that ZmBAG8 negatively regulates drought tolerance in maize by influencing stomatal aperture to enhance water loss.

### ZmSKIP interacts with ZmSnRK2.3

**Fig 5:**
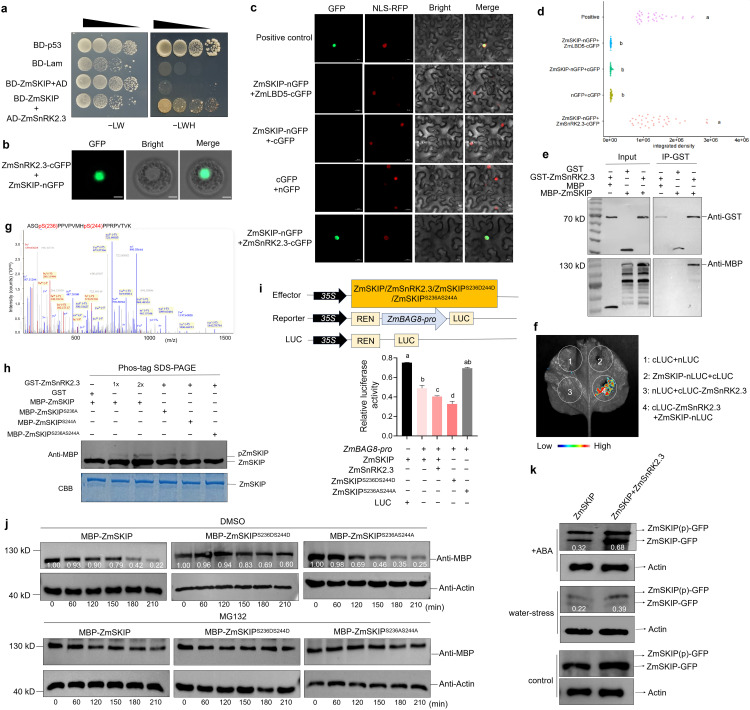
ZmSKIP is phosphorylated by ZmSnRK2.3 at Ser^236^ and Ser^244^ to increase its inhibitory effect on Zm*BAG8.* a. Y2H assay of ZmSKIP protein interacting with ZmSnRK2.3, with pGBKT7(BD)-p53 as the positive control and BD-lam as the negative control. 1. pGADT7(AD)+BD-ZmSKIP as the negative control. 2. Yeast transformants streaked on -LW medium (SD/-Trp/-Leu) and -LWH medium (SD/-Trp/-Leu/-His/-Ade), respectively. b. Bimolecular Fluorescent Complementation (BiFC) assays for the ZmSKIP and ZmSnRK2.3 interaction in maize protoplast. Bar = 10 μm c. BiFC assays for the ZmSKIP-ZmSnRK2.3 interaction and control plasmids in *Nicotiana benthamiana* leaves. The overlay of the grayscale and BiFC fluorescence is shown in the nucleus. Positive control: pXYn106-LBD33 and pXYc104-LBD5. NLS: nucleus marker. nGFP: the empty vector of pXYn106. cGFP: the empty vector of pXYc104. Bar = 20 μm. d. The GFP fluorescence intensity in **c** which analyzed by ImageJ. More than 30 nucleus were analyzed for each group of GFP fluorescence values, and the background was removed. e. Pull-down assay of ZmSKIP protein interacting with ZmSnRK2.3 protein *in vitro*. GST-ZmSnRK2.3 protein was incubated with MBP-ZmSKIP protein *in vitro*, using MBP protein as a control. The precipitated proteins were analyzed by immunoblotting with anti-GST or anti-MBP antibody. f. Luciferase complementation imaging (LCI) assays confirm the interaction of ZmSKIP with ZmSnRK2.3. The indicated constructs were co-transformed in *Nicotiana benthamiana* leaves. Luciferase signals were imaged at 72 h post-transfection. The color represents the luciferase signal in each group. All experiments were repeated three times with similar results. g. The phosphorylation sites of ZmSKIP identified using the CO-IP/MS assay. h. *In vitro* kinase activity assay of ZmSnRK2.3 phosphorylating the ZmSKIP protein. Intact MBP-ZmSKIP or site-mutated MBP-ZmSKIP^S236A^, MBP-ZmSKIP^S244A^, or MBP-ZmSKIP^S236AS244A^ incubated with GST-ZmSnRK2.3 protein was detected by an anti-MBP antibody by Phos-tag mobility shift assays. Coomassie (CBB)-stained total protein was used as the loading control. i. Dual-LUC assay of ZmSKIP and ZmSnRK2.3 on *ZmBAG8* promoter activity in maize protoplast. The relative LUC activities were normalized to the reference REN LUC. The mean value and standard deviation (SD) were calculated from three biological replicates. The LUC activity derived from mutant effectors ZmSKIP^S236AS244A^, ZmSKIP^S236D244D^, ZmSnRK2.3, and ZmSKIP. j. ZmSKIP protein degradation assay *in vitro*. MBP-ZmSKIP protein abundance was detected using an anti-MBP antibody. ACTIN was used as an internal control. DMSO was used as a negative control for MG132. 0–210 min represents the incubation times of GST-ZmBAG8 and MBP-ZmSKIP protein. The relative intensity of each MBP band normalized to the control (Actin band) is shown below. k. Western blot analysis of the phosphorylation level of ZmSKIP by Anti-GFP using Phos-tag PAGE. ZmSnRK2.3 and ZmSKIP were co-injected into tobacco and dehydrated for 6h or 50 μM ABA treatment. ZmSKIP alone was used as the negative control. ACTIN was used as an internal control. The relative intensity of each GFP band normalized to the control (actin band) is shown below. Different letters show significant differences (P < 0.05) between them.

Interestingly, we observed that another similar-sized protein band appeared near the ZmSKIP target band in *ZmSKIP* OE transgenic plants under drought stress. After treatment with λ-protein phosphatase, the upper band disappeared or became weak (S6a Fig), and thus, we speculated that ZmSKIP may be phosphorylated under drought stress. A yeast two-hybrid (Y2H) assay was conducted to identify protein kinase that interacts with ZmSKIP using the ZmSKIP protein without transcriptional activation activity as bait to screen the library of maize (B73) leaf cDNAs (S6b Fig). Over 200 individual colonies and a total of 188 valid sequencing data were obtained, which encompassed 103 different proteins (S6 Data). Two protein kinases of SNF1-related protein kinase 2, serine threonine protein kinase 3 (ZmSnRK2.3, Zm00001d029975) and calcium-dependent protein kinase 27 (ZmCPK27, Zm00001d012457) were identified in the library. The Y2H verification assays demonstrated that ZmSnRK2.3 (Fig 5a), not ZmCPK27 (S6c Fig), could interact with ZmSKIP in yeast cells. These results indicate that ZmCPK27 might be a false positive result, and ZmSnRK2.3 was selected for further research. Similarly, a bimolecular fluorescence complementation (BiFC) assays in maize protoplast (Fig 5b) and *Nicotiana benthamiana* leaves (Fig 5c and S7 Data) both confirmed the interaction of ZmSnRK2.3 with ZmSKIP in the nucleus *in vivo*. The addition of the positive and negative controls and analysis of GFP fluorescence intensity further confirmed this result (Fig 5c-5d). Furthermore, pull-down assays demonstrated that glutathione S-transferase (GST)-tagged ZmSnRK2.3 could bind to MBP-tagged ZmSKIP *in vitro* (Fig 5e). In luciferase complementation imaging (LCI) assays, LUC fluorescence signals were observed in *Nicotiana benthamiana* leaves co-infiltrated with cLUC-ZmSnRK2.3 and ZmSKIP-nLUC (Fig 5f). Taken together, these results indicate that ZmSKIP physically interacts with ZmSnRK2.3 in the nucleus.

### ZmSKIP is phosphorylated by ZmSnRK2.3 at Ser^236^ and Ser^244^ to increase its inhibitory effect on Zm*BAG8*

To test whether ZmSnRK2.3 could phosphorylate ZmSKIP, we performed a Phos-tag mobility shift assay [49] and immunoprecipitation (Co-IP/MS) assays to examine the phosphorylation site of ZmSKIP in maize leaves using an anti-FLAG antibody (S8 Data). We identified two phosphorylation sites in ZmSKIP: Ser^236^ and Ser^244^ (Fig 5g). The Phos-tag mobility shift assay results showed MBP-ZmSKIP as a single and faster migrating band in the absence of GST-ZmSnRK2.3; however, a second, slower migrating band gradually appeared with increasing amounts of GST-ZmSnRK2.3, indicating that ZmSKIP was phosphorylated by ZmSnRK2.3 *in vitro* (Fig 5h). Furthermore, we prepared ZmSKIP proteins with two intact phosphorylation sites (MBP-ZmSKIP), with either site mutated (MBP-ZmSKIP^S236A^, MBP-ZmSKIP^S244A^), or both sites mutated (MBP-ZmSKIP^S236AS244A^), and incubated them with GST-ZmSnRK2.3 to assess *in vitro* kinase activity. The results showed that when MBP-ZmSKIP^S236AS244A^ was incubated with GST-ZmSnRK2.3, the slowly shifting bands disappeared, demonstrating that ZmSKIP was phosphorylated by ZmSnRK2.3 at Ser^236^ and Ser^244^ (Fig 5h). Additionally, Dual-LUC assays were performed to determine the significance of ZmSKIP phosphorylation. When expressing ZmSKIP in maize protoplast, LUC activity derived from the *ZmBAG8pro*:LUC reporter was significantly decreased compared to that of the control (ZmSKIP alone). When ZmSnRK2.3 was added additionally, the inhibitory effect on *ZmBAG8pro*:LUC was further enhanced. LUC activity derived from the mutant effectors ZmSKIP^S236AS244A^ was comparable to that of the ZmSKIP effector alone. In contrast, the mutant effector ZmSKIP^S236D244D^ enhanced the inhibition of *ZmBAG8* expression (Fig 5i). To investigate the mechanisms underlying ZmSKIP phosphorylation, how triggers an increase in the transcriptional inhibitory activity of *ZmBAG8*, ubiquitination degradation analysis was performed. In WT maize cells, MBP-ZmSKIP, mutant MBP-ZmSKIP^S236DS244D^, and MBP-ZmSKIP^S236AS244A^ proteins were treated with DMSO or MG132 (control). MBP-ZmSKIP^S236DS244D^ exhibited lower degradation rates, whereas MBP-ZmSKIP^S236AS244A^ showed higher degradation rates than MBP-ZmSKIP protein (Fig 5j). When treated with MG132, the degradation rate of all the proteins did not change significantly. These results reveal that ZmSnRK2.3 phosphorylates ZmSKIP at Ser^236^ and Ser^244^ by enhancing its protein stability to increase its inhibitory effect on *ZmBAG8*.

The kinase activity of SNRK2-class proteins is often activated in ABA-dependent drought response pathways [50,^51^]. To determine whether the phosphorylation of ZmSKIP by ZmSnRK2.3 is more likely to occur under drought stress and ABA-dependent, ZmSnRK2.3-RFP and ZmSKIP-GFP were co-infiltrated in Nicotiana benthamiana leaves and further treated with drought stress and ABA. Then, the phosphorylation level of ZmSKIP was analyzed using a GFP antibody. The more obvious phosphorylated ZmSKIP was detected when both ZmSnRK2.3 and ZmSKIP were expressed under drought stress and ABA treatment compared to ZmSKIP alone or under normal conditions (Fig 5k). Additionally, we investigated ZmSnRK2.3 expression under drought stress conditions. *ZmSnRK2.3* expression was increased in both Ac7643 (drought-tolerant inbred line, abbreviated as P1) and Ac7729/TZSRW (drought-sensitive inbred line, abbreviated as P2) maize under PEG treatment compared to that in the controls (S7a Fig), and this was similar to the results obtained for the natural maize population (S7b Fig). We concluded that ZmSnRK2.3 responds to drought stress and activates its protein kinase activity, leading to the phosphorylation of ZmSKIP at Ser^236^ and Ser^244^, thereby increasing its protein stability. This enhancement amplified the inhibitory effect of ZmSKIP on the target gene *ZmBAG8* under drought stress in maize.

### ZmSKIP could be recruited by ZmBAG8 in SGs to decrease its protein abundance

**Fig 6:**
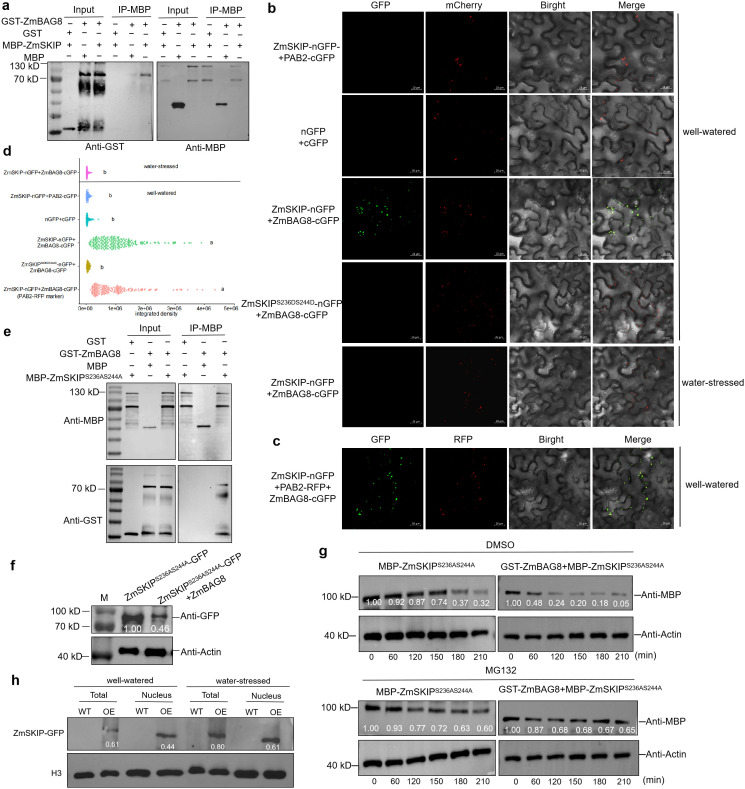
ZmSKIP interacts with ZmBAG8 in SGs. a. Pull-down assay of ZmSKIP protein interacting with ZmBAG8 protein *in vitro*. GST-ZmBAG8 protein was incubated with MBP-ZmSKIP protein *in vitro*, using MBP protein as a control. The precipitated proteins were analyzed by immunoblotting with anti-GST or anti-MBP antibody. Additional ~70 KDa band in anti-MBP section is a non-specific binder. b. BiFC assays for the ZmSKIP, ZmSKIP^S236DS244D^ and ZmBAG8 interactions under well-watered or water-stressed conditions in *N. benthamiana* leaves. The fluorescence signal of mcherry and the signal of eGFP overlapped in the stress granules (SGs). eiF3: the SG marker. nGFP: the empty vector of pXYn106. cGFP: the empty vector of pXYc104. Bar = 20 μm. The ZmSKIP-nGFP + PAB2-cGFP and nGFP + cGFP groups were used as negative controls. c. BiFC assays for the ZmSKIP and ZmBAG8 interactions under well-watered condition in *N. benthamiana* leaves. The fluorescence signal of RFP and the signal of eGFP overlapped in the stress granules (SGs). PAB2: the SG marker. Bar = 20 μm. d. The GFP fluorescence intensity in **b-c** which analyzed by ImageJ. More than 50 SGs were analyzed for each group of GFP fluorescence values, and the background was removed. e. Pull-down assay of ZmSKIP^S236AS244A^ protein interacting with ZmBAG8 protein *in vitro*. MBP- ZmSKIP^S236AS244A^ protein was incubated with GST-ZmBAG8 protein *in vitro*, using GST protein as a control. The precipitated proteins were analyzed by immunoblotting with anti-MBP or anti-GST antibody. Additional ~70 KDa band in anti-MBP section is a non-specific binder. f. Co-expression of ZmBAG8 and ZmSKIP^S236AS244A^-eGFP proteins in the leaf cells of tobacco. Anti-eGFP analysis of the protein level of ZmSKIP^S236AS244A^ by western blot only showed expression of ZmSKIP^S236AS244A^ as a control and Actin as an internal control. The relative intensity of each eGFP band normalized to the control (Actin band) is shown below. g. ZmSKIP protein degradation assay *in vitro*. MBP-ZmSKIP^S236AS244A^ protein abundance was detected using an anti-MBP antibody. ACTIN was used as an internal control. MG132 was used as a negative control for DMSO. 0–210 min represents the incubation times of GST-ZmBAG8 and MBP-ZmSKIP^S236AS244A^ protein. The relative intensity of each MBP band normalized to the control (Actin band) is shown below which analyzed by ImageJ. h. Analysis of protein abundance of ZmSKIP in the nucleus of maize. WT, wild type; OE, ZmSKIP overexpression transgenic line; Total, the total protein of maize; Nucleus, the nuclear component proteins after cytoplasmic-separation assays. H3 was used as internal control. Relative intensity of each ZmSKIP-GFP band normalized to the control (H3 band) is shown below which analyzed by ImageJ.

Notably, ZmBAG8 was also identified in Co-IP/MS assays (S8 Data). To confirm whether ZmSKIP interacts with ZmBAG8, we first performed a pull-down assay. The results revealed that the recombinant MBP-ZmSKIP was pulled down by GST-ZmBAG8 *in vitro* (Fig 6a). We further performed a BiFC assay to determine whether ZmSKIP interact with ZmBAG8 *in vivo*. Strong GFP signals were detected in *N. benthamiana* leaves co-infiltrated with ZmSKIP-nGFP and ZmBAG8-cGFP constructs, which co-localized with two stress granules (SGs) marker proteins eIF3 (Fig 6b and S9-S13 Data files) or POLY(A) BINDING PROTEIN 2 (PAB2) (Fig 6c and S14 Data), indicating that ZmSKIP could interact with ZmBAG8 *in vivo*. The addition of the negative control and analysis of GFP fluorescence intensity further confirmed this result (Fig 6d). Notably, we observed that the interaction between ZmSKIP and ZmBAG8 disappeared under drought stress (Fig 6b), indicating that their interaction is unique under normal conditions. Additionally, we found that the strong green fluorescence was not located in the nucleus but was instead observed in locations similar to the ZmBAG8 or ZmSKIP phosphorylation loss proteins (ZmSKIP^S236A^, ZmSKIP^S244A^, or ZmSKIP^S236AS244A^) that were expressed in SGs (S8a-S8b Fig). Pull-down assays further demonstrated that mutant ZmSKIP^S236AS244A^ protein indeed interacted with ZmBAG8 (Fig 6e), which did not apply to ZmSKIP^S236DS244D^ protein (Fig 6b). In addition, we detected whether this interaction because of the subcellular localization of ZmSKIP altered under drought stress. The subcellular localization of intact and unphosphorylated ZmSKIP did not change under either drought or normal conditions (S8b-S8c Fig). The quantitative fluorescence intensity of ZmSKIP nucleic vs cytosol protein under normal and drought conditions further illustrate its function by interaction with ZmBAG8 (S8d Fig). Therefore, we concluded that unphosphorylated ZmSKIP could be recruited by ZmBAG8 to SGs, and this interaction is not dependent on drought stress.

To explore the biological significance of the interaction between ZmSKIP^S236AS244A^ and ZmBAG8, these proteins were expressed in tobacco, and their levels were analyzed. The results revealed that when ZmSKIP^S236AS244A^ and ZmBAG8 were co-injected, the protein abundance of ZmSKIP^S236AS244A^ was significantly reduced compared with that of ZmSKIP^S236AS244A^ alone (Fig 6f). Ubiquitination degradation analysis was further performed. In WT maize cells, the MBP-ZmSKIP^S236AS244A^, GST-ZmBAG8, and MBP-ZmSKIP^S236AS244A^ proteins were treated with DMSO or MG132 (control). The GST-ZmBAG8 and MBP-ZmSKIP^S236AS244A^ combination exhibited an increased degradation rate and decreased protein aboundance of MBP-ZmSKIP^S236AS244A^ compared to that of the alone MBP-ZmSKIP^S236AS244A^ group. When MG132 was added, ZmSKIP showed a slight instability, but there was no significant difference in the degradation rates and protein aboundance between the two groups (Fig 6g). The slight instability of ZmSKIP might be due to the incorporation of a large amount of MG132. In order to further confirm the above results, we also analyzed the protein abundance of ZmSKIP under drought stress in maize. The results showed that the total and nuclear components content of ZmSKIP protein were significantly increased under drought stress, compared to the normal conditions (Fig 6h). These results indicate that under normal conditions, ZmBAG8 recruits unphosphorylated ZmSKIP to SGs to decrease its protein abundance in the nucleus, so as to increase the *ZmBAG8* transcription. Under drought stress, ZmBAG8 does not interact with ZmSKIP, therefore, the ZmSKIP protein in the nucleus increases, which further enhances the inhibitory effect of *ZmBAG8* transcription.

## Discussion

Drought is a major abiotic stress factor that limits plant growth, development, and productivity worldwide [8,48,52,53]. Plants cope with drought stress primarily via root morphogenesis and stomatal movement. Under drought conditions, plants alter their root growth and architecture to enhance water and nutrient absorption while closing their stomata to reduce water loss [52,53]. Stomata as the vital organs for exchanging gas and water between the plant and external environment, play critical roles in the activities of plant life by ensuring maximum absorption of CO_2_ for photosynthesis, and meanwhile controlling the optimal transpiration. Drought stress affects not only the light reactions, but also the assimilation efﬁciency of the dark reactions, thereby reducing the photosynthetic products. Photosynthesis products further determines the plant biomass production [22]. In this study, we found that ZmSKIP positively regulates drought tolerance by modulating stomatal aperture in maize. The reduced stomatal aperture of *ZmSKIP* OE transgenic plants led to decreased transpiration rates, stomatal conductance, and water loss rates, resulting in enhanced drought tolerance under WS conditions (Figs 1, 2). Notably, *ZmSKIP* OE transgenic plants and *skip-aa Mu* mutants exhibited no changes in the stomatal density and root-shoot ratio compared to the controls (WT and *skip-AA*) (S2 Fig). Interestingly, the photosynthetic rate of the *ZmSKIP* OE transgenic plants shows no variation, but the plant height was significantly decreased (Figs 1, 2). GO analysis of RNA-seq results showed that the DEGs were mainly enriched in the jasmonic acid biosynthesis and the long-chain fatty acid metabolism pathway which might be the main reason for the variation in plant height and yield of *ZmSKIP* OE transgenic plants [54,55]. Another possible reason is that ZmSKIP caused the narrower stomatal aperture, the lower water loss, so the metabolic balance has been changed under drought stress, which are likely to cause the changes of cell wall morphology and cell cycle, and then lower plant growth [53,56]. Furthermore, ZmSKIP suppresses the transcription activity of *ZmBAG8* through the “TAATA” motif (Fig 3e). Under normal condition, the stomatal aperture of *bag8* mutants were slightly larger than that of the control, however, *bag8* mutants exhibited a smaller stomatal aperture phenotype similar to that of *ZmSKIP* OE plants under drought stress. This might be the dynamic adjustment of the stomatal opening to stabilize the balance of CO_2_ and water intake and outflow to keep its growth and drought tolerance (Fig 4). Taken together, these findings suggest that ZmSKIP enhances maize drought tolerance by reducing the stomatal aperture, likely through inhibiting *ZmBAG8* expression.

Drought stress is often accompanied by the production of phytohormones, such as ABA and ethylene, which induce stomatal closure. ROS are recognized as secondary messengers during stomatal opening and closure [8,48]. ABA is sensed by the abscisic acid receptor proteins pyrabactin resistance 1-like (PYLs) [57,58]. ABA-bound PYLs inhibit clade A protein phosphatase type 2Cs (PP2Cs), thereby preventing them from suppressing SnRK2s. ABA-activated SnRK2s phosphorylate TFs, such as ABA-responsive element-binding factors (ABFs), and these phosphorylated ABFs regulate the expression of ABA-responsive genes to enhance drought tolerance [50,59]. In this study, we observed that ZmSKIP responded to ABA and ethylene, and the ROS content was significantly altered in *ZmSKIP* transgenic plants (S3 Fig). Additionally, under drought stress, ABA-activated ZmSnRK2.3 phosphorylates ZmSKIP at Ser^236^ and Ser^244^ to increase its protein stability, which further suppresses the expression of the stomatal aperture-related gene *ZmBAG8* to enhance drought tolerance in maize (Fig 5). These results indicate that ZmSKIP responds to drought stress, possibly through the ZmSnRK2.3-dependent ABA signaling pathway, promoting stomatal closure to decrease ROS accumulation and water loss, ultimately improving drought tolerance in maize.

SKIP proteins have been extensively identified in diverse organisms ranging from yeast to humans. In plants, SKIP functions as a bifunctional regulator, serving both as a splicing factor by interacting with spliceosome components and as a transcriptional activator by regulating target gene expression [16,17,20,60]. Zhang et al. [29] demonstrated that SKIP binds to the pre-mRNAs of ABA signaling-related genes, including PYL7/8, ABI1/5, and HAB, to regulate their splicing under abiotic stress. As a transcriptional coregulator, SKIP forms functional complexes with key developmental regulators. For instance, it interacts with STM to establish the SKIP-STM transcriptional complex, which coordinates the expression of SAM-related genes, such as CLAVATA3 (CLV3) and GA2OX1, in *Arabidopsis* [26]. In addition, SKIP associates with the Paf1c complex to regulate flowering by activating *FLC* transcription [23]. However, the specific binding sites and direct target genes that mediate the SKIP-regulated drought responses in maize remain poorly understood. Our study reveals that ZmSKIP functions as a transcription factor that directly binds to the “TAATA” conserved motif within the *ZmBAG8* promoter (Fig 3), thereby enhancing drought tolerance through *ZmBAG8* regulation in maize. These findings provide a crucial theoretical basis for understanding the mechanisms underlying SKIP-mediated drought resistance in plants.

Compared with the extensive and in-depth research on BAGs in animals, research examining the BAG family in plants is relatively scarce and has mainly focused on plant growth, autophagy, and stress responses [33,40]. In animals, BAG proteins are predominantly located in the nucleus, whereas in plants, they function in both the cytoplasm and nucleus. Under salt stress, OsBAG4 acts as a bridge by interacting with OsSUVH7 and OsMYB106 to activate *OsHKT1;5* expression and reduce salt sensitivity in the nucleus [61]. The RING-type E3 ubiquitin ligase (EBR1) directly targets OsBAG4 for ubiquitin-mediated cytoplasmic degradation [62]. In the endoplasmic reticulum (ER), under normal conditions, AtBAG7 binds to AtBiP2 and AtbZIP28. Under heat stress conditions, unfolded proteins accumulate and AtBAG7 is SUMOylated and proteolytically cleaved, enabling it to translocate into the nucleus. AtBAG7 interacts with WRKY29 to induce its transcription of *AtBAG7* [63]. Here, we revealed that ZmBAG8 functions as a chaperone co-regulator and is exclusively expressed in the SGs of maize (S8a Fig). The cytoplasmic ZmBAG8 protein can recruit ZmSKIP to SGs and reduce the abundance of ZmSKIP in the nucleus via the 26S proteasome pathway (Fig 6). Combining the results of the subcellular localization of unphosphorylated ZmSKIP under drought stress and the BiFC assays of ZmBAG8 and ZmSKIP^S236DS244D^, we conclude that ZmBAG8 can recruit unphosphorylated ZmSKIP^S236AS244A^ to SGs, and this interaction disappears under drought stress. This was a unique interaction between ZmSKIP and ZmBAG8 under normal conditions. ZmSKIP and ZmBAG8 regulate each other’s expression at the transcriptional and protein levels, respectively, thereby achieving a dynamic balance of their expression in plants. This is a novel functional mechanism of the SKIP and BAG proteins.

**Fig 7:**
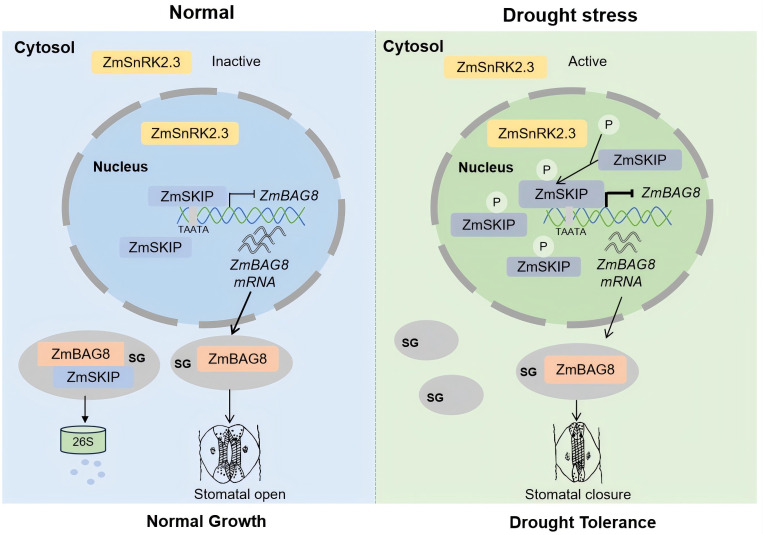
Schematic model depicting the detailed molecular mechanism of ZmSKIP regulating drought tolerance through control of the stomatal aperture in maize. ZmSKIP suppresses *ZmBAG8* expression through the “TAATA” motif*.* Under normal conditions, ZmSKIP protein is recruited by ZmBAG8 to SGs to reduce its protein abundance in the nucleus, so as to further decrease *ZmBAG*8 suppression in the nucleus. ZmBAG8 positively regulates the stomatal aperture and maintains normal growth for maize. Under drought stress, ZmSnRK2.3 kinase activity increases, resulting in an increased phosphorylation level of ZmSKIP to enhance its stability. Then, the phosphorylated ZmSKIP further increases its inhibitory effect on *ZmBAG8*, resulting in the lower stomatal aperture and the higher drought resistance of maize. Arrows indicate facilitation, short vertical lines indicate inhibition. The thickness of the lines reflects the strength of the inhibiting or promoting effect.

We determined a working model for ZmSKIP in maize drought stress response (Fig 7). ZmSKIP suppresses *ZmBAG8* expression through the “TAATA” motif*.* Under normal conditions, ZmSKIP is recruited by ZmBAG8 to SGs, which reduces its abundance in the nucleus (Fig 6), so as to further decrease *ZmBAG*8 suppression. ZmBAG8 positively regulates stomatal aperture and maintains normal growth in maize. Under drought stress, the kinase activity of ZmSnRK2.3 increases, resulting in an elevated phosphorylation level of ZmSKIP at Ser^236^ and Ser^244^, thereby enhancing its stability. Phosphorylated ZmSKIP aggregated in the nucleus, further strengthening its inhibitory effect on *ZmBAG8*. These results led to a lower stomatal aperture and water loss and higher drought resistance in maize. Drought tolerance refers to the ability of plants to sustain a certain level of physiological activities under severe drought stress conditions through the regulation of thousands of genes and series of metabolic pathways to reduce or repair the resulting stress damage. In our studies, under drought stress conditions, the increased ROS level which induced by ABA promotes the activation of ZmSnRK2:3. ZmSnRK2:3 further regulates the expression of Zm*SKIP* and Zm*BAG8*, thereby controlling the stomata closure. ZmSKIP provides a yield advantage under drought stress conditions with reduced plant height and stomatal aperture. The reduced stomatal aperture maybe lead to the lower water loss, so the metabolic balance has been changed under drought stress, further casuse the changed cell wall morphology, shortened growth cycle and declined plant growth and leaf area. This might be a morphological regulatory mechanism developed by the plants to adapt to drought stress, not any adverse effect on the plant growth after the change in stomatal aperture [56]. Not only *ZmSKIP* gene, the other genes or loci related to drought tolerance identified in this study may also be valuable for improving traits in maize and other crops.

## Materials and methods

### Plant materials and growth conditions

Maize (*Zea mays L.*) inbred line KN5585 was used as the wild-type (WT) for phenotypic experiments and all genetic transformations involving *ZmSKIP-*overexpressing (OE) transgenic plants. WT and transgenic seeds were placed on germination paper and incubated at 28 °C until the radicle and germ developed. The seeds were cultured in vermiculite until they possessed two leaves and were then transferred to nutrient soil for growth at 28 °C under 14 h light/10 h dark cycle. Large pots (length × width × height = 54 × 28 × 14 cm^3^) were used for the drought stress test of seedlings grown together in one pot. Transgenic and WT plants were grown in the same pots. Drought stress was managed by controlled irrigation at the 3-leaf stage. Photographs were taken and the survival rate and water loss rate of the isolated leaves was measured when the seedlings displayed mild wilting under drought stress (the soil water content <8%).

For field trials, *ZmSKIP* OE transgenic plants and *skip-aa* mutants were cultivated in Hainan (N: 18.3902°, E:109.1966°) from 2021 to 2023 (three replicates per year). The average planting density was approximately 60,000 ha^-1^. The field trials were employed with two treatment groups: drought and normal. It was a double-row layout with a row length of 3 meters. Each treatment group had three replicates. In the field, the plants were planted back-to-back, with observation paths 80–100 cm wide. In the normal group, the total water (sum of rainfall and irrigation water) supply for the entire growth period of maize was generally maintained at 5,500 m^3.^ha^-1^. In the drought stress group, sufficient irrigation was provided at sowing to ensure full seedling emergence, and no irrigation was carried out after the ten-leaf stage, and the final soil moisture content < 8%. The soil moisture content was measured using the four-in-one soil moisture meter (TR-6D, Shunkeda, Beijing), with the average value taken from three points at different locations in the soil. Plant height was measured 15 d after pollination, and yield data were measured when the plants matured under drought stress. At least 14 plants of each line are used for data analysis.

### Generation of *ZmSKIP* OE transgenic plants and *skip Mu* insert mutants

DNA fragments encoding FLAG and eGFP tags were fused to ZmSKIP by PCR and cloned into pCAMBIA3301 between the BamHI and SacI restriction sites. Its expression is driven by the maize ubiquitin promoter which containing in pCAMBIA3301 vector. Successful generation of all constructs was confirmed by sequencing. The *skip* Mu insert mutants which the background is inbred line B73 were purchased from the ChinaMu Project website (http://chinamu.jaas.ac.cn/Default.html). PCR was used to screen for *skip* Mu insert mutants. qPCR was used to detect the *ZmSKIP*-OE transgenic plants (S15 Data). Homozygous T3 progenies of *skip* mutants were generated by self-crossing and used in subsequent experiments.

### RNA extraction and real-time qPCR

To analyze the expression patterns of *ZmSKIP* in different tissues, total RNA was extracted from different samples using the RNAprep Pure Polysaccharide Polyphenol Plant Total RNA Extraction Kit (TIANGEN, Beijing, China) according to the manufacturer’s instructions. Approximately 1–2 µg of total RNA was reverse transcribed into cDNA using Hiscript III Reverse Transcriptase (Vazyme, Nanjing, China) according to the manufacturer’s instructions. The cDNAs were used as templates for qPCR with gene-specific primers, and the polyubiquitin gene GAPDH was used as a reference. Each qPCR was performed in triplicate and the gene-specific primers used in this analysis are listed in S15 Data.

### Measurement of stomatal aperture and conductance

At the three-leaf stage, the middle parts of the second fully expanded leaf were used for stomatal measurements by four persons. The first person was responsible for fixing the stomata at different time points using nail polish. The second one took images and measured data by optical microscope (Leica, Germany) and ImageJ software (National Institutes of Health, Bethesda, Maryland, USA). Stomatal aperture size refers to the distance between guard cell walls. The third person analyzed data and the last one which not participating in the project provided the unmasking key of genotypes. The same samples at different time points (10:00 AM, 11:00 AM, 15:00 AM, 16:00 PM, 17:00 PM) and genotype-masked methods were used for stomatal conductance measurement by an LI-6800 portable photosynthesis system (Li-Cor, Lincoln, NE, USA). Three replicates were used, each containing more than 50 stomata of six plants.

### Measurement of the water loss from detached leaves

To measure the rate of water loss via transpiration, the third fully expanded leaf was excised from five independent plants per genotype. Immediately after excision, the cut ends were sealed with petroleum jelly to prevent moisture loss from the wound site, ensuring that water loss occurred primarily through stomata and cuticle. The leaves were then placed in a plate on the laboratory bench and weighed at designated time intervals (0 h and 12 h). The proportion of fresh weight lost was calculated based on the initial weight of the plant. Three biological replicates were performed for each measurement [64].

### RNA-seq analysis

The coleoptile from *ZmSKIP*-OE (OE2) and WT maize were employed. Each genotype contained nine plants with similar germination stage was separated into three biological replicates for RNA-seq analysis. RNA-seq libraries were constructed and sequenced on an Illumina NovaSeq 6000 platform by OE Biotech (Shanghai, China). Raw gene-level read counts were obtained from feature-based quantification files and processed in R (v4.1.0). For each sample, the gene ID and the raw read count column were extracted, and count tables from all samples were merged by gene ID to generate a unified count matrix. Differential expression analysis between the OE2 and WT was performed using the DESeq2 package. Differential expression between the two samples was assessed using Wald tests. Resulting P values were adjusted for multiple testing using the Benjamini–Hochberg false discovery rate (FDR) method. Genes with an absolute log2 fold change greater than 1 and an adjusted P value (FDR) < 0.05 were defined as significantly differentially expressed genes(DEGs). Genes with positive log2 fold changes were classified as up-regulated, whereas those with negative log2 fold changes were classified as down-regulated. Volcano plots were generated using ggplot2 to visualize differential expression results. DEGs with FDR < 0.05 were selected for Gene Ontology (GO) enrichment analysis using GOATOOLS [65]. Significantly enriched GO terms were identified based on Bonferroni-corrected P values with a significance threshold of P < 0.05. Maize genome sequence (B73 Genome v4.0) was used for the all analysis.

### Candidate gene identification of the rate of water loss from detached leaves

The generalized linear model regression analysis was applied to associate gene expression levels in seedling leaves under drought stress [47] with the rate of water loss measured in detached leaves in R (version 4.4.1). P-values of the model were adjusted for multiple testing using the Benjamini-Hochberg (BH) procedure. To screen for high-confidence candidate genes with robust phenotypic contributions and stable expression patterns across the population, we applied the following three filtering criteria: (1) Phenotypic variance contribution: the *R*^2^ value was derived from the model deviance to represent the proportion of phenotypic variance explained by the gene expression. The *R* ^2^ of each gene was calculated using the formula: *D*^2^ = 1-*D*_model_/*D*_null_, where *D*_model_ corresponds to the residual deviance of the fitted model and *D*_null_ corresponds to the null deviance. Only genes with an *D*^2^ > 0.15 were retained. (2) Differential expression significance: since sample duplicates were lacked among the population, Student’s *t*-*t*ests were used to compare gene expression between WW and WS conditions. Genes were required to show highly significant differential expression with a *t*-*t*est FDR < E^-10^. (3) Expression stability: to ensure consistent regulation across the diversity panel, we calculated the SD of the expression difference (WS-WW) for each gene across all inbred lines. Genes with an SD < 10 were selected, indicating stable expression changes among the different inbred lines.

### Gene ontology (GO) enrichment analysis

GO enrichment analysis was performed to elucidate the biological functions of the identified candidate genes. Gene IDs were mapped to Entrez IDs based on the Zea mays B73 RefGen_v4 annotation. GO enrichment analysis was conducted using the enrichGO function in the clusterProfiler R package [66]. Enrichment results were visualized using the ggplot2 package in R (version 4.4.1). Enrichment score was defined as the ratio of the proportion of input genes annotated to a specific term (GeneRatio) to the proportion of background genes annotated to that term (BgRatio). Top enriched terms were displayed in bubble plots, where the x-axis represents the Enrichment Score, the size of the dot corresponds to the number of candidate genes (Gene Count), and the color gradient represents the statistical significance, scaled as -log_10_(FDR).

### Yeast two-hybrid (Y2H) assays

To investigate which proteins interact with ZmSKIP, the full-length coding region of ZmSKIP was cloned into the pGBKT7 (BD) vector to screen the cDNA library of maize coleoptiles. First, BD-fused ZmSKIP was mixed with empty pGADT7 (AD) and co-transformed into the Y2H Gold yeast strain to test its self-activating activity. Owing to the lack of self-activating activity of ZmSKIP (S6b Fig), Y2H library screening experiments were conducted. We also performed reverse validation experiments on candidate proteins in which full-length coding regions were cloned into AD vectors. Yeast cells harboring BD-ZmSKIP and AD candidate protein vectors were serially diluted and grown on non-selective (SD/-Trp-Leu, -LW) or selective (SD/-Trp-Leu-His-Ade, -LWH) media. BD-p53 was used as a positive control (+) and BD-Lam was used as a negative control (-).

### Bimolecular fluorescence complementation (BiFC) assays

For the BiFC assays, the full-length coding regions (CDS) of ZmSKIP, and ZmSnRK2.3 and ZmBAG8 were individually cloned into the pXYc106 and pXYn104 vectors and fused with the N-terminus (nYFP) and C-termini (cYFP) of YFP. Thereafter, the indicated plasmid combinations were cotransformed into *Agrobacterium* cells and transiently expressed in *Nicotiana benthamiana* leaves by a non-project team member. Another person is responsible for the image acquisition through confocal laser microscopy (LSM800; Zeiss, Jena, Germany). During images acquisition, all groups used the same parameters to avoid overexposure. The third member collected the fluorescence signal intensity using ImageJ software. Cell organelles were segmented by manual masking or automated thresholding, and only organelles with moderate expression levels (gated by fluorescence intensity) were included (excessive or too dark fluorescence were excluded to avoid false positives), and background subtraction was performed in 2–3 non-cellular area. At least 30 nucleus or 50 SGs of each group were analyzed by the the fourth person who are not the project member. Finally, the genetypes were decrypted by the first person. The positive control plasmids of pXYn106-LBD33 and pXYc104-LBD5 were provided by Feng et al. [67]. ZmSKIP and ZmLBD5 (which located in nucleus), ZmSKIP and cGFP, and cGFP and nGFP groups were used as negative controls. The same plasmids of ZmSKIP and ZmSnRK2.3 were co-transformed into maize protoplasts, which performed according to Maize Protoplasts Preparation and chemical transformation Kit (Coolaber PPT141, Beijing, China). Meanwhile, the same gene-construct-masked techniques were used of BiFC assays between ZmSKIP and ZmBAG8. ZmSKIP and PAB2 (which located in stress granules), and cGFP and nGFP groups were used as negative controls. PAB2-RFP of the SG marker was performed by Wu et al. [68]. The primers and constructs used are listed in S15 Data.

### Transient dual-luciferase expression assays

Reporters were constructed using the pGreenII 0800-LUC vector and effectors were constructed using the pCAMBIA2300-eGFP vector. An approximately 2,000-bp promoter fragment of *ZmBAG8* was amplified and cloned into pGreenII 0800-LUC to drive the LUC reporter. ZmSnRK2.3 and ZmSKIP were cloned into pCAMBIA2300-eGFP vectors and driven by the *CaMV35S* promoter. Empty vector pCAMBIA2300-eGFP was used as a control. The primers and constructs used are listed in S15 Data. Transient dual-luciferase assays were performed on maize protoplasts, which performed according to Maize Protoplasts Preparation and chemical transformation Kit (Coolaber PPT141, Beijing, China). A dual-luciferase assay kit (Vazyme, DL101–01, China) was used to measure luciferase activity. Three biological replicates were used for each experiment.

### Pull down assays

ZmSKIP was fused to a Maltose Binding Protein (MBP) tag, and ZmSnRK2.3 and ZmBAG8 were fused to Glutathione S-transferase (GST) tags. MBP-ZmSKIP protein was produced in *Escherichia coli* strain BL21 (WeiDi, Shanghai, China) with the addition of 0.5 mM isopropyl β-D-1-thiogalactopyranoside (IPTG) at 37 °C and then purified using Maltose gel beads (NEB, USA). The purified MBP-ZmSKIP protein was incubated with glutathione sepharose (GE Healthcare) that was combined with different GST fusion proteins (GST-ZmSnRK2.3 and GST-ZmBAG8) for 2–3 h at 4 °C, respectively. The beads were then washed thrice with 1 × phosphate-buffered saline (PBS). Proteins were eluted from the beads with 100 µL elution buffer (1 × PBS + 10 mM glutathione + 50 mM Tris-Cl, pH 8.0) and loaded onto the SDS–PAGE gel. Gel blots were analyzed using an anti-MBP antibody (working dilution 1:2,000; Abcam, UK) and anti-GST antibody (working dilution 1:2,000; Abcam, UK). An empty GST tag was used as a negative control.

### CUT&Tag assays

CUT&Tag assays were performed as described by Tao et al. [69], with the following modifications. Briefly, 1 g of leaf tissue per sample was ground and resuspended in 30 mL of nuclear isolation buffer (10 mM Tris-HCl pH 8.0, 10 mM KCl, 0.5 mM spermidine, 14 mM β-mercaptoethanol, 0.5% Triton X-100, and 1 × protease inhibitor cocktail) through two layers of Mira-cloth and then centrifuged at 600 × *g* for 5 min to collect the precipitates. Then, the pellet was washed three times using nuclear wash buffer (10 mM Tris-HCl pH 8.0, 150 mM NaCl, 0.5 mM spermidine, and 1 × protease inhibitor cocktail), and the nuclei were resuspended in 1 mL of antibody buffer (50 mM Tris-HCl PH 6.0, 1 mM EDTA, 150 mM NaCl, 0.5 mM spermidine, 1 mg/ml BSA, 0.05%w/v digitonin, and 1 × protease inhibitor cocktail). Anti-FLAG antibody or IgG control antibody (1–2 μg) was used for each immunoprecipitation reaction. Transposase insertion and DNA library construction were performed according to the manufacturer’s instructions using the Hyperactive Universal CUT & Tag Assay Kit (Vazyme #TD903) and TruePrep Index Kit V2 (Vazyme #TD202). A Qubit fluorescence quantifier was used to detect library concentration and quality. Paired-end Illumina sequencing was performed on the barcoded libraries using an Illumina HiSeq 2500 or another massive parallel DNA sequencer following the manufacturer’s instructions.

### Chromatin immunoprecipitation-quantitative polymerase chain reaction (ChIP-qPCR) analysis

ChIP-qPCR assay was performed following the protocol described earlier [70]. Leaves from *ZmSKIP* OE transgenic plants were collected for these analysis. After quickly frozen in liquid nitrogen and grinding, the powders were transferred to nuclear isolation buffer (10 mM HEPES pH 7.6, 1 M sucrose, 5 mM KCl, 5 mM MgCl_2_, 5 mM EDTA, 1% [w/v] PVP, 14 mM β-mercaptoethanol, 0.6% [v/v] Triton X-100, and 1 × protease inhibitor cocktail) containing formaldehyde at room temperature for 10-min cross-linking, and then 2 M glycine was added to stop the cross-linking. After isolation and sonication, chromatin complexes in the leave samples were incubated with 20 µg anti-GFP, at the same time, the sample added the same amount of lgG antibody was used as negative control (mock). Specific primers were used to analyze the interactions of ZmSKIP and *ZmBAG8* promoter. Three biological replicates were performed for each test, and the Tukey HSD test of 1-way ANOVA was used for statistical analysis (significant difference: P < 0.05). All of the primers used are listed in S15 Data.

### Electrophoretic mobility shift assays (EMSA)

Complementary oligonucleotides were synthesized and annealed to double-stranded DNAs (95 °C for 5 min and then slowly cooled to RT and transferred to 4 °C), and *ZmBAG8* DNA probes were incubated with 300 ng of purified ZmSKIP protein in binding buffer (50 mM KCl, 1 mM DTT, 5 mM MgCl_2_, 2.5% [v/v] glycerol, and 10 mM Tris-HCl, pH 7.5) for 30 min at 4 °C. DNA–protein complexes were separated by 4% (w/v) non-denaturing PAGE on ice. Biotin-labeled EMSA was performed according to the manufacturer’s instructions using a Chemiluminescent Nucleic Acid Detection Module Kit (0020158; Thermo Scientific, Rockford, IL, USA). For the competition assay, 1-fold and 2-fold amounts of unlabeled DNA fragments (competitors) or mutated conserved binding sequences (mprobes) were added to the reactions. All oligonucleotides used in the EMSA are listed in S15 Data.

### In vitro phosphorylation assays

In vitro phosphorylation assays were performed as described previously [71]. Briefly, 3 mg of recombinant ZmSKIP and 10 mg of recombinant ZmSnRK2.3 proteins were mixed in a 20 µL reaction (25 mM Tris–HCl, pH 7.5, 1 mM EGTA, 20 mM MgCl_2_, 1 mM dithiothreitol, 200 µM ATP, and 1 × phosphatase inhibitor) and incubated at room temperature for 30 min. The reaction was stopped by adding 5 × SDS loading buffer and incubating at 95 °C for 10 min. Samples were separated on SuperSep Phos-tag SDS–PAGE gels (Wako 193–16711), and then analyzed by immunoblotting with an anti-MBP antibody (working dilution 1:2,000; Abcam, UK).

### Transcriptome-wide association study (TWAS)

The expression data (FPKM) of maize leaves at seedling stage were from Sun et al. [47]. To identify genes whose expression levels were significantly correlated with the leaf water loss rate, a transcriptome-wide association study was conducted. A linear model was fit individually for each phenotype-gene combination, in which the explanatory variable is the expression value of a gene across individuals [72]. To control for the false positive rate derived from multiple hypothesis testing (typically involving thousands of genes), the p values were adjusted using the Benjamini-Hochberg (FDR) procedure. Genes with an FDR-adjusted p value < 0.05 were considered significantly associated with leaf water loss rate [73].

### Statistical analysis

Data are presented as the mean ± standard deviation unless otherwise specified. Statistical significance was determined by one- or two-way analysis of variance (ANOVA) corrected with Tukey’s multiple comparison test or Student’s *t*-test using GraphPad Prism version 9.0 software (GraphPad Software, San Diego, CA, USA). Different letters represent significant differences (P < 0.05). *t-*t*est, with P < 0.05,**t-tes*t*, with P < 0.01, and ***t-test, wi*t*h P < 0.001. Density analysis of the western blots was conducted using the ImageJ software.

## Supporting information

S1 Fig Characterization of the expression pattern and the relationship between ZmSKIP and drought tolerance in maize. (TIFF)

S2 Fig Phenotypic analysis of *ZmSKIP* transgenic maize at the seedling stage. (TIF)

S3 Fig Plant hormone response to ZmSKIP and reactive oxygen species staining in *ZmSKIP* transgenic maize. (TIF)

S4 Fig RNA-seq analysis of the differentially expressed genes (DEGs) in the coleoptile of ZmSKIP-OE transgenic maize. (TIF)

S5 Fig Characterization of the expression pattern and its relationship with ZmBAG8 drought tolerance in maize. (TIF)

S6 Fig Characterization of ZmSKIP protein. (TIF)

S7 Fig Characterization of the expression pattern in ZmSnRK2.3 in response to drought. (TIF)

S8 Fig Analysis of the subcellular localization of ZmBAG8 and mutated ZmSKIP proteins. (TIF)

S1 Data The water loss rate of 98 inbred lines in maize. (XLS)

S2 Data The stomatal aperture of *ZmSKIP* transgenic plants. (XLS)

S3 Data The yield of *ZmSKIP* transgenic plants. (XLS)

S4 Data Differentially expressed genes (DEGs) in the maize coleoptile libraries were identified using RNA-seq analysis (*ZmSKIP* OE2 vs. WT). (XLS)

S5 Data The stomatal aperture of *bag8* mutant plants. (XLS)

S6 Data Proteins identified by yeast two-hybrid library analysis using ZmSKIP protein. (XLS)

S7 Data The BiFC images included in Fig 5c. (RAR)

S8 Data Proteins identified in *ZmSKIP*-OE transgenic maize by Co-IP analysis using anti-FLAG antibody. (XLS)

S9 Data The BiFC images in Fig 6b of ZmSKIP-nGFP + PAB2-cGFP group. (RAR)

S10 Data The BiFC images in Fig 6b of nGFP + cGFP group. (RAR)

S11 Data The BiFC images in Fig 6b of ZmSKIP-nGFP + ZmBAG8-cGFP water-stressed group. (RAR)

S12 Data The BiFC images in Fig 6b of ZmSKIP^S236DS244D^-nGFP + ZmBAG8-cGFP group. (RAR)

S13 Data The BiFC images in Fig 6b of ZmSKIP-nGFP + ZmBAG8-cGFP well-watered group. (RAR)

S14 Data The BiFC images included in Fig 6c. (RAR)

S15 Data Primers and probes used in the experiments. (XLS)
