# Supplementary figures and images for: ZmSKIP enhances drought tolerance by reducing stomatal aperture in maize
Source: PLoS Genet. 2026 Apr 13;22(4):e1012077. doi: 10.1371/journal.pgen.1012077 (PMC13075797; doi:10.1371/journal.pgen.1012077)

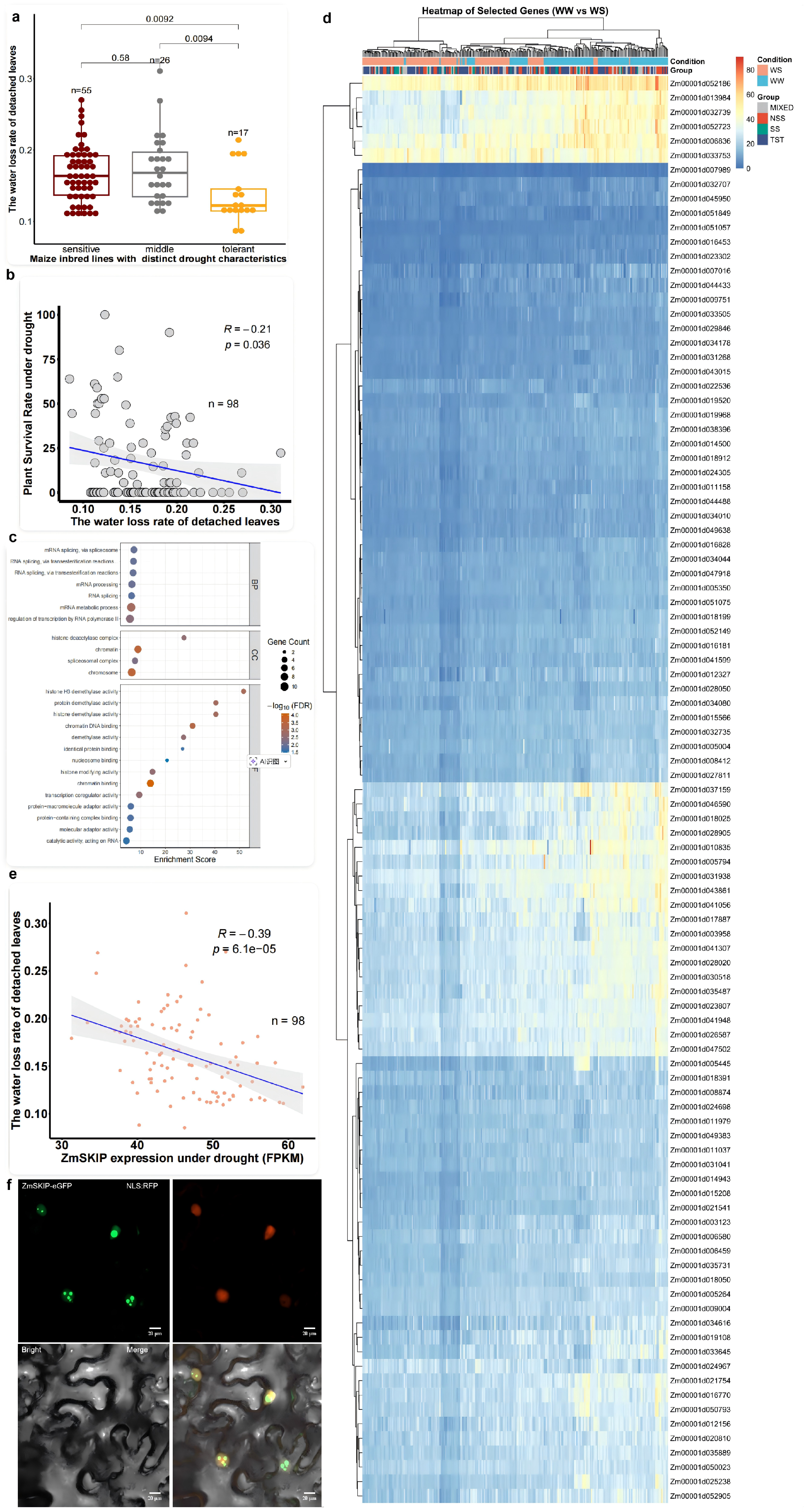

Supplement: S1 Fig — (TIFF) [file pgen.1012077.s001.tiff]

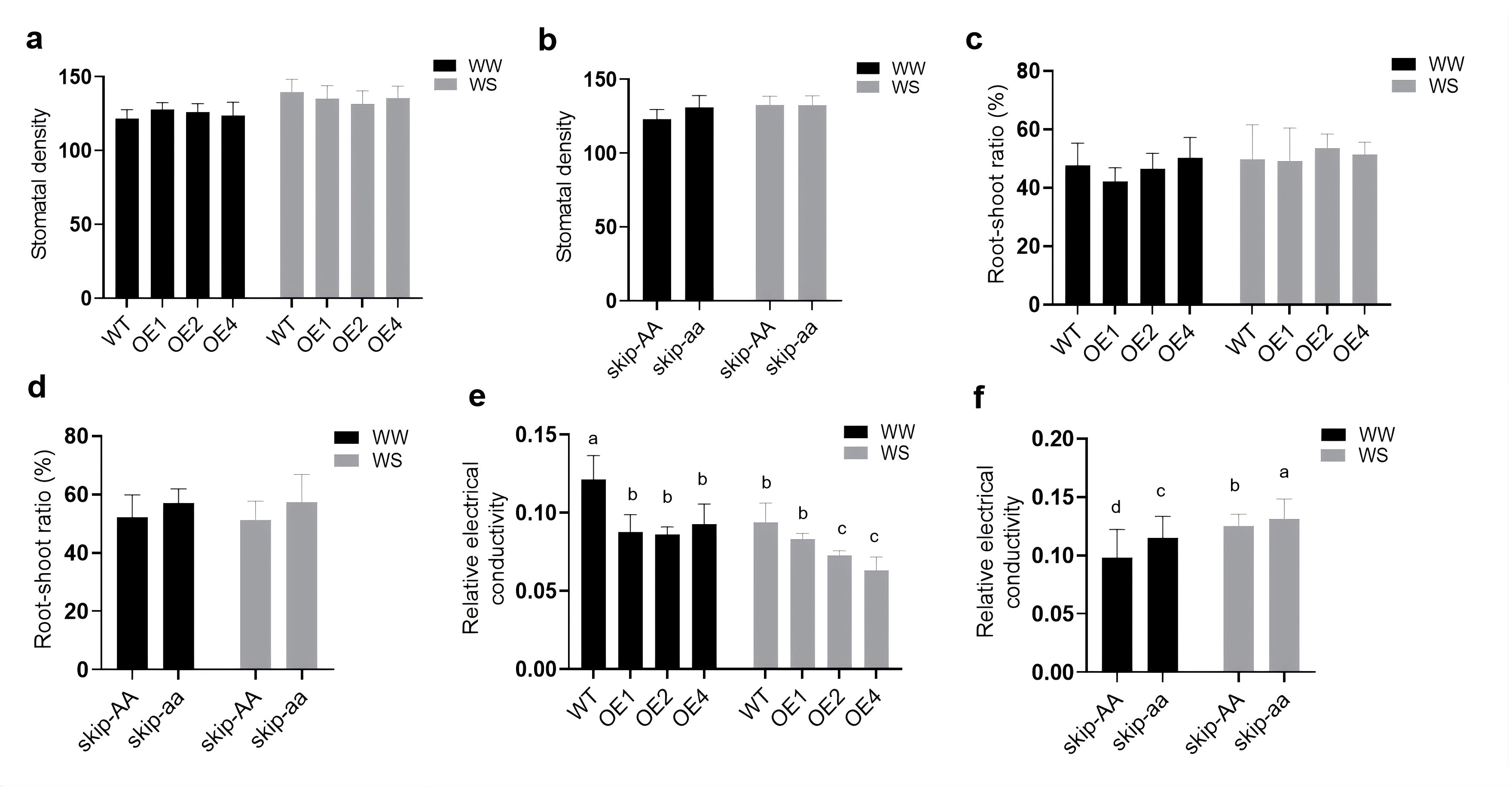

Supplement: S2 Fig — (TIF) [file pgen.1012077.s002.tif]

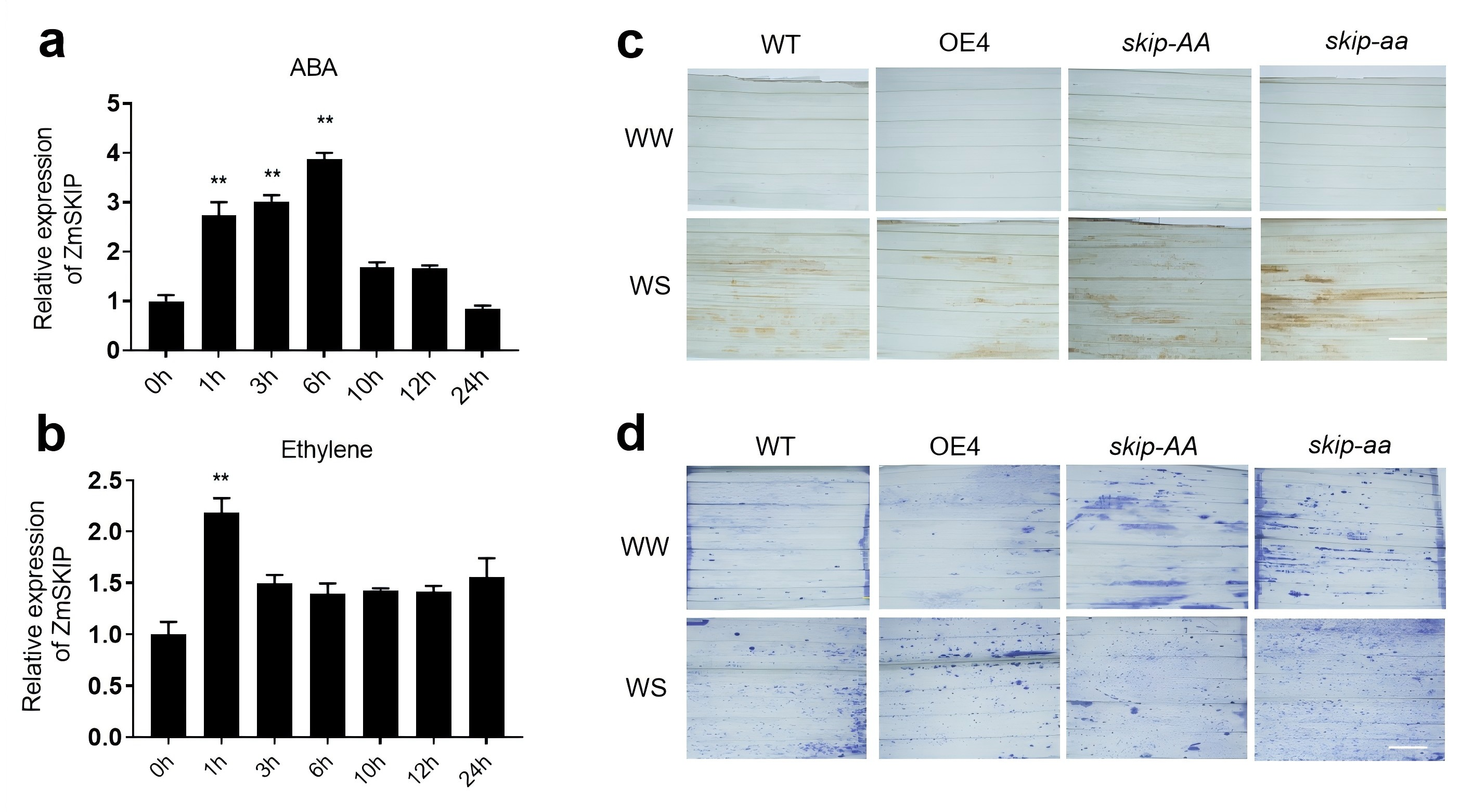

Supplement: S3 Fig — (TIF) [file pgen.1012077.s003.tif]

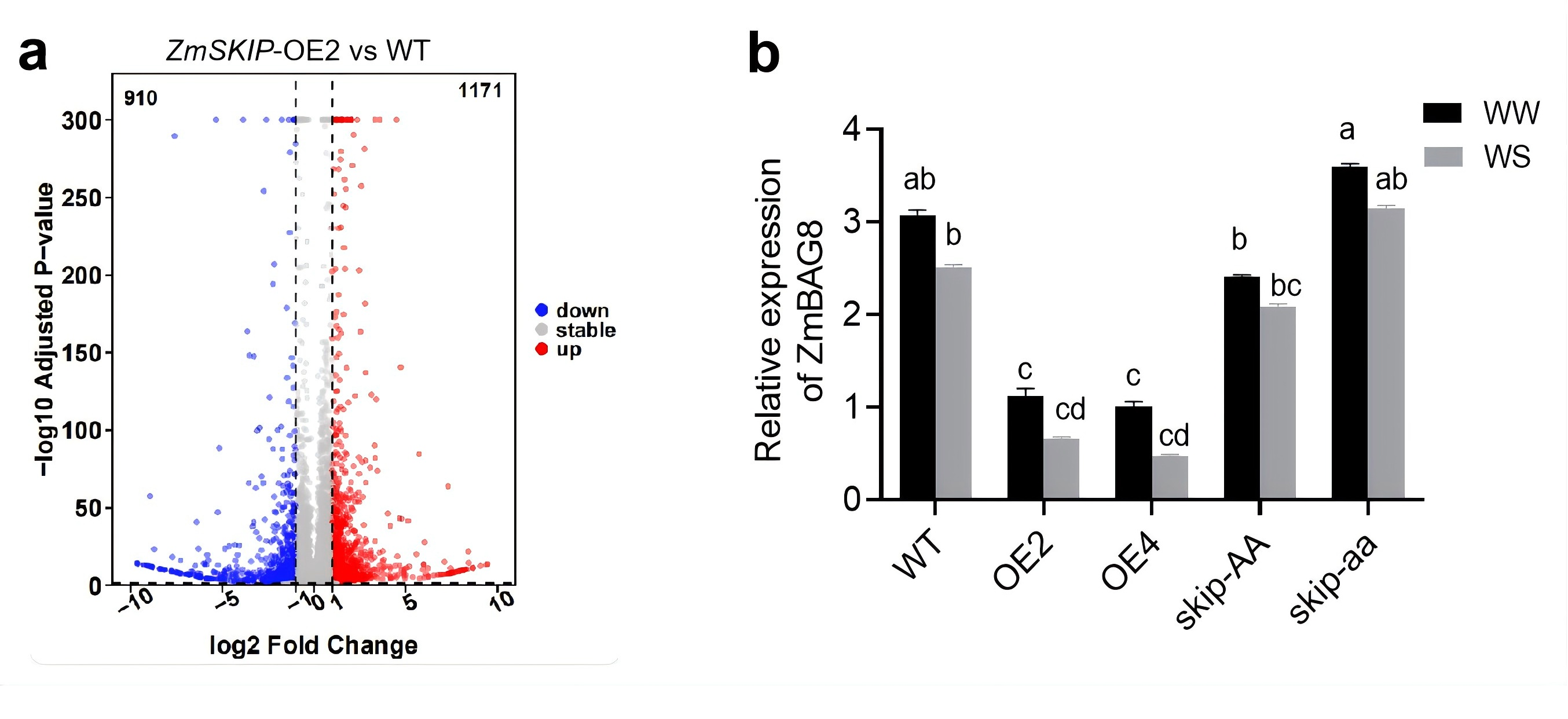

Supplement: S4 Fig — (TIF) [file pgen.1012077.s004.tif]

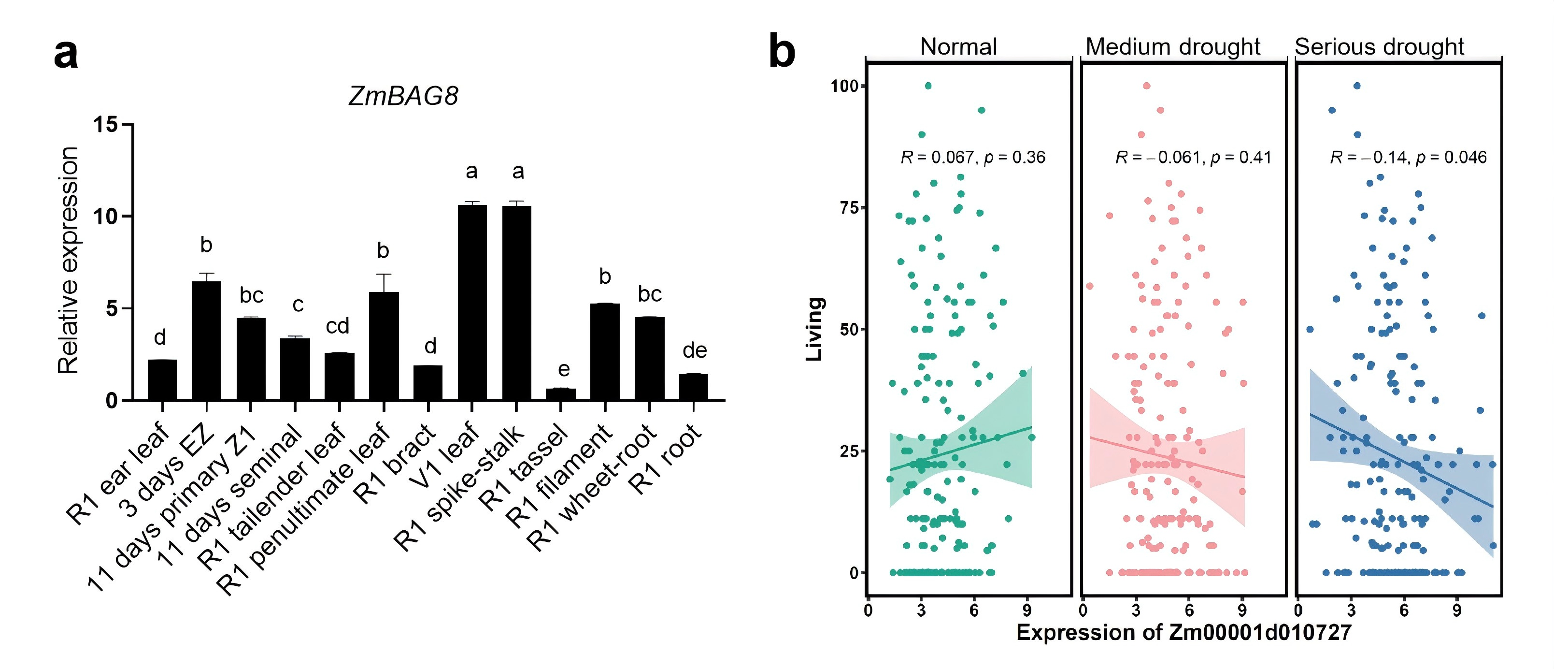

Supplement: S5 Fig — (TIF) [file pgen.1012077.s005.tif]

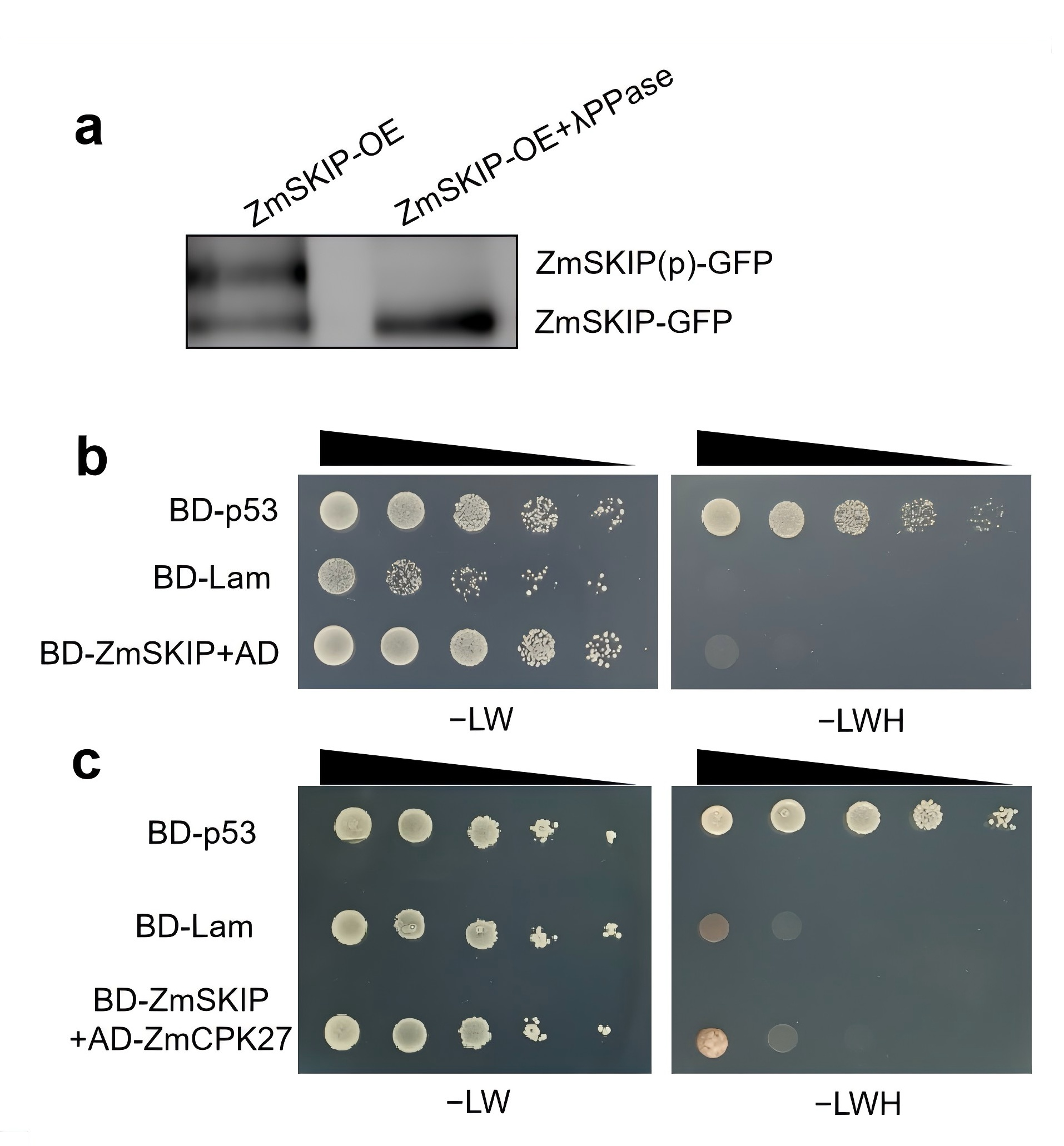

Supplement: S6 Fig — (TIF) [file pgen.1012077.s006.tif]

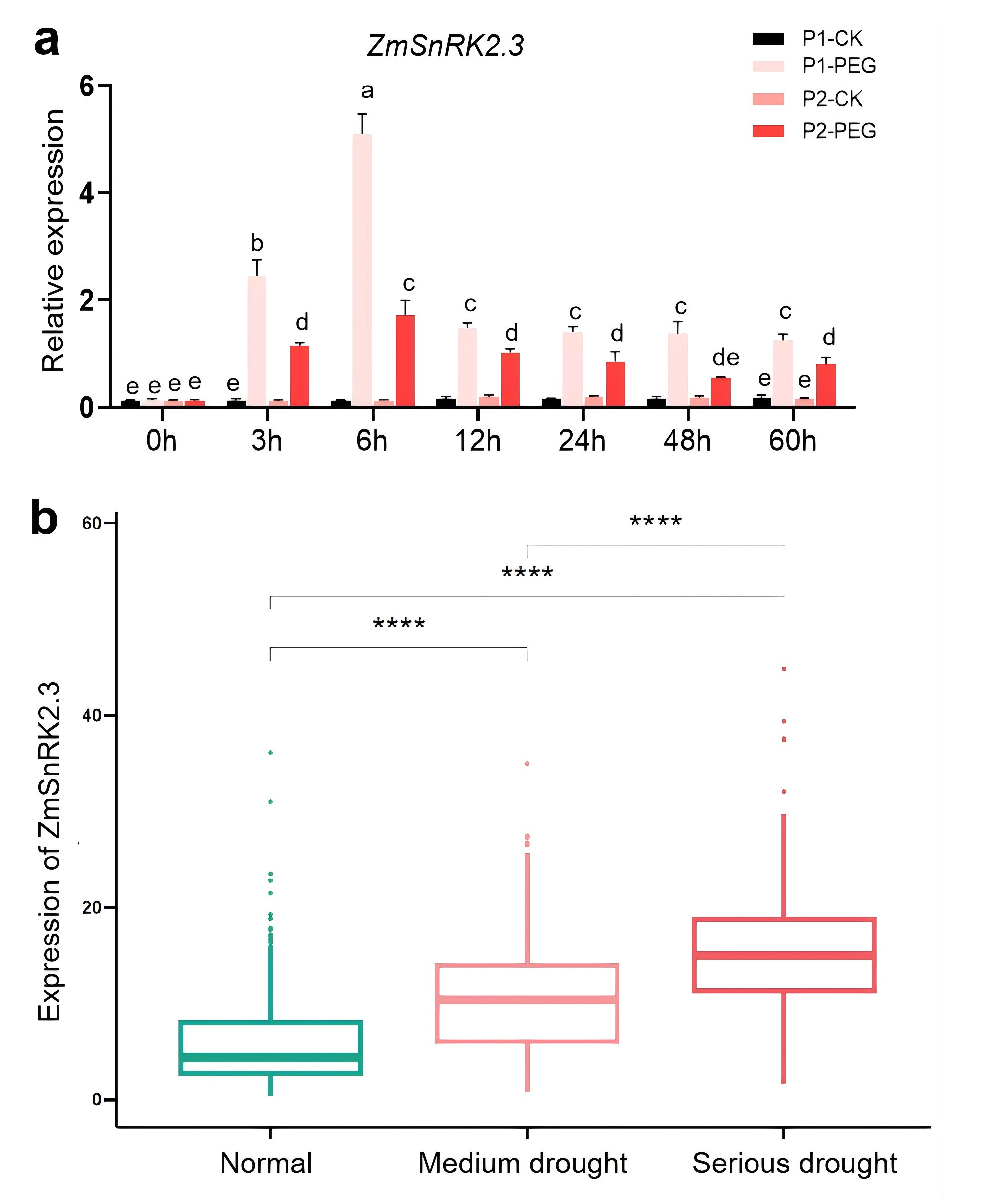

Supplement: S7 Fig — (TIF) [file pgen.1012077.s007.tif]

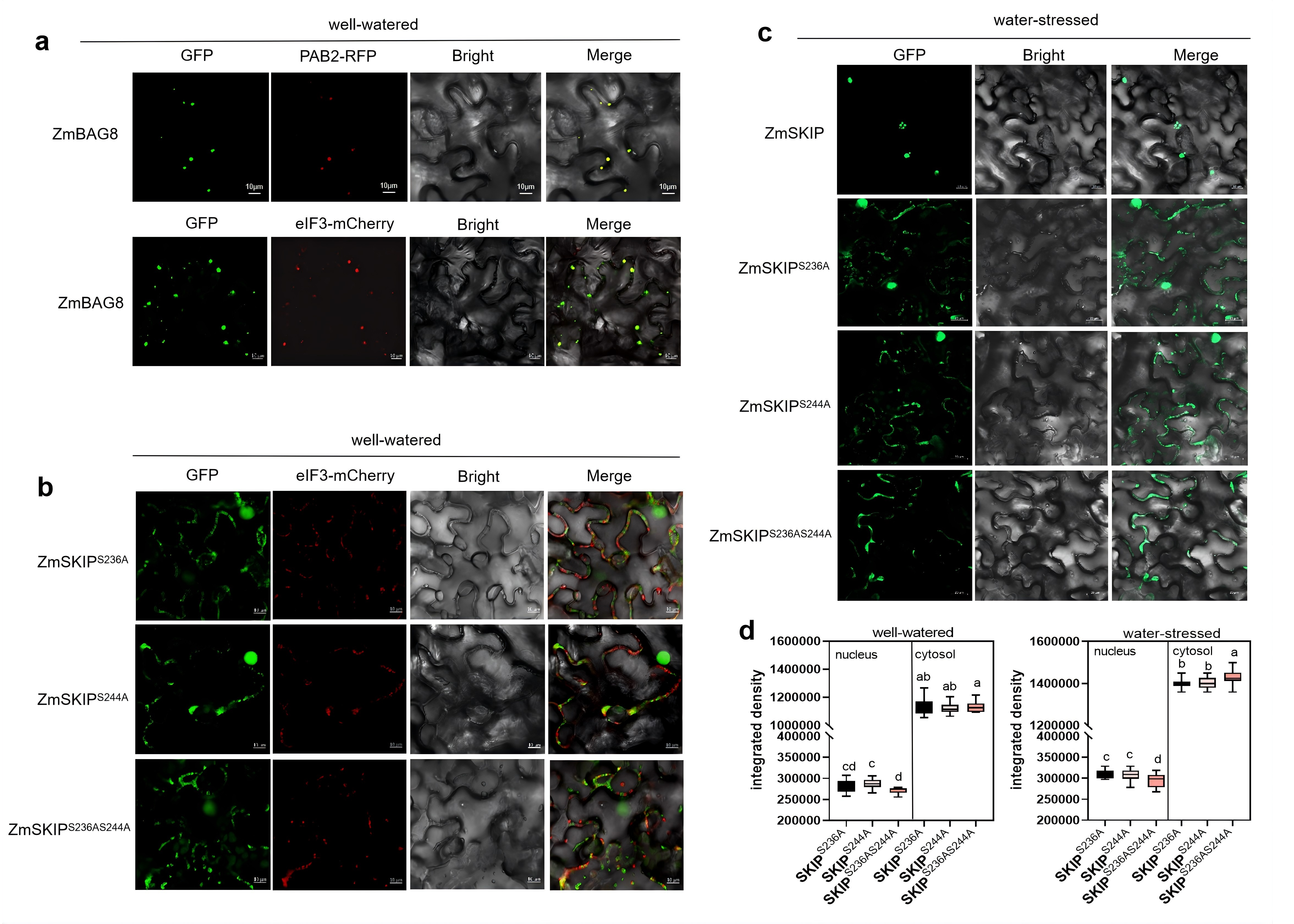

Supplement: S8 Fig — (TIF) [file pgen.1012077.s008.tif]
